# Patterns of the within-host evolution of human norovirus in immunocompromised individuals and implications for treatment

**DOI:** 10.1016/j.ebiom.2024.105391

**Published:** 2024-10-12

**Authors:** Ray W. Izquierdo-Lara, Nele Villabruna, Dennis A. Hesselink, Claudia M.E. Schapendonk, Sol Ribó Pons, David Nieuwenhuijse, Jenny I.J. Meier, Ian Goodfellow, Virgil A.S.H. Dalm, Pieter L.A. Fraaij, Jeroen J.A. van Kampen, Marion P.G. Koopmans, Miranda de Graaf

**Affiliations:** a Department of Viroscience, Erasmus University Medical Center, Rotterdam, the Netherlands; b Erasmus MC Transplant Institute, Rotterdam, the Netherlands; c Division of Virology, Department of Pathology, University of Cambridge, UK; d Department of Internal Medicine, Division of Allergy & Clinical Immunology; Department of Immunology, Erasmus University Medical Center Rotterdam, the Netherlands

**Keywords:** Norovirus, Chronic infections, Immunocompromised, Virus evolution, Immunoglobulin, Antigenic evolution

## Abstract

**Background:**

Currently, there is no licensed treatment for chronic norovirus infections, but the use of intra-duodenally-delivered immunoglobulins is promising; nevertheless, varying results have limited their wide use. Little is known about the relationship between norovirus genetic diversity and treatment efficacy.

**Methods:**

We analyzed the norovirus within-host diversity and evolution in a cohort of 20 immunocompromised individuals using next-generation sequencing (NGS) and clone-based sequencing of the capsid (VP1) gene. Representative VP1s were expressed and their glycan receptor binding affinity and antigenicity were evaluated.

**Findings:**

The P2 domain, within the VP1, accumulated up to 30-fold more non-synonymous mutations than other genomic regions. Intra-host virus populations in these patients tended to evolve into divergent lineages that were often antigenically distinct. Several of these viruses were widely resistant to binding-blocking antibodies in immunoglobulin preparations. Notably, for one patient, a single amino-acid substitution in the P2 domain resulted in an immune-escape phenotype, and it was likely the main contributor to treatment failure. Furthermore, we found evidence for transmission of late-stage viruses between two immunocompromised individuals.

**Interpretation:**

The findings demonstrated that within-host noroviruses in chronic infections tend to evolve into antigenically distinct subpopulations. This antigenic evolution was likely caused by the remaining low immunity levels exerted by immunocompromised individuals, possibly undermining antiviral treatment. Our observations provide insights into norovirus (within-host) evolution and treatment.

**Funding:**

Erasmus MC grant mRACE, the European Union's 10.13039/501100007601Horizon 2020 research and innovation program under grant agreement No. 874735 (VEO), and the NWO STEVIN award (Koopmans).

**Research in context**

**Evidence before this study**

Albeit typical norovirus infections are acute, immunocompromised individuals can develop chronic infections, where the virus tends to accumulate mutations over time and diverge from commonly-circulating strains. We searched PubMed with key words (“norwalk” [tiab] OR “norovirus” [tiab]) AND (“persistent” [tiab] OR “chronic” [tiab] OR “immunocompromised” [tiab] OR “immune compromised” [tiab]) AND “evolution” for publications in English published up to February 2024 and accessed relevant articles. Current evidence shows that during chronic infections, mutations in the major capsid (VP1) gene are common, particularly in the P2 domain, yet there was no statistical evidence that this region is subject to more changes than other genomic regions. It is known that norovirus in chronic infections can be genetically divergent, and it has recently been reported that within-host evolution can result in distinct subpopulations. However, there is a lack of experimental evidence showing the effects of this genetic diversity on the virus phenotype. There is no specific antiviral drug available to treat norovirus infections, but the off-label use of immunoglobulin (Ig) preparations is promising. Nonetheless, varying results are reported in literature, and the reasons of this variability have not been determined. A better understanding of how (intra-host) virus diversity affects treatment is needed to improve therapeutic strategies. Immunocompromised individuals have been proposed as potential reservoirs of novel virus variants. However, there is no evidence of human-to-human transmission of late-stage noroviruses from immunocompromised to other individuals.

**Added value of this study**

Using the most extensive set of samples from chronic norovirus infections to date, we have determined some of the common evolutionary characteristics of the virus and provided insights into how viral diversity affects treatment. Whole-genome analysis of chronic infections revealed that the P2 domain accumulated the most non-synonymous mutations over time, followed by the p22 and VP2 genes. The relative high number of patients compared with previous studies allowed us to determine mutational hotspots of the VP1, showing that residues in known antigenic sites are the most variable during chronic infections. Analysis of the quasispecies showed that norovirus subpopulations emerge as lineages that frequently display marked phenotypic differences. Evidence from binding-blocking assays showed that intra-host viruses can be antigenically distinct to each other and to community-circulating viruses. Moreover, these viruses are often widely resistant to blocking antibodies in Ig preparations, potentially undermining Ig treatment. A single mutation was sufficient to confer resistance to Ig treatment, but also reduced binding affinity to glycan receptors. Interestingly, this mutation was present as a minor variant before treatment and became transiently dominant after treatment. Additionally, we found evidence for the transmission of late-stage viruses between two immunocompromised individuals.

**Implications of all the available evidence**

This study demonstrates that during chronic infections, phenotypically distinct viruses arise within the viral population, which can directly affect antiviral treatment efficacy. Our findings together with previous studies show that intra-host norovirus populations tend to evolve into lineages. Here we determined that norovirus preferentially acquires more non-synonymous mutations in specific regions of the genome, especially in antigenic sites of the VP1. This suggests that the main mechanism of norovirus within-host evolution in chronic infections is the low immune-pressure exerted by the host. We hypothesize that this can favor the emergence of viral (sub-)populations harboring resistance-associated mutations, explaining the cases where Ig treatment failed, even if these escape mutations have a fitness cost (e.g. low glycan binding affinity). The role of immunocompromised individuals as reservoirs of novel virus variants is yet to be determined. A potential role is supported by the similarities between the within-host evolution in chronic infections and the observed for community-circulating viruses, as well as late-stage virus transmission between immunocompromised individuals. However, currently there is no evidence of outbreaks in the community caused by late-stage chronic noroviruses. Given that persistent infections in immunocompromised hosts are observed for many viruses, that do not typically cause such infections, the implications of this study go beyond noroviruses.

## Introduction

Norovirus is the leading cause of gastroenteritis, and most affected clinically are children, elderly and immunocompromised individuals.^1^ Norovirus infection and its associated illness can be remarkably persistent and severe in immunocompromised individuals.^2^^,^^3^ Currently, no specific antiviral drug is licensed to treat acute or chronic norovirus infections. Several therapeutic strategies have been used, including tampering of immunosuppressive therapy and off-label administration of drugs, such as favipiravir, nitazoxanide (NTZ), ribavirin (Rbv), interferon-alpha (IFNa) and immunoglobulin (Ig) therapy.^4^, ^5^, ^6^ However, these strategies have shown variable results,^5^^,^^7^, ^8^, ^9^, ^10^ potentially due to strain diversity and inter-patient variation. Moreover, in chronic infections, noroviruses tend to be genetically divergent from those circulating in the community, often accumulating mutations along the genome,^2^^,^^11^, ^12^, ^13^ potentially making the virus less susceptible to these therapeutic strategies. Therefore, it is fundamental to understand the virus evolutionary patterns within these patients and their effects on treatment.

The human norovirus genome contains three open reading frames (ORFs) that encode eight viral proteins.^1^ ORF1 encodes the non-structural proteins p48, NTPase, p22, VPg, 3Cpro, and the RNA-dependent RNA-polymerase (RdRp). ORF2 and ORF3 encode the major (VP1) and minor (VP2) capsid proteins.^1^ The VP1 encompasses: The Shell (S) and Protruding (P) domains, of which the latter has the P2 sub-domain that contains the histo-blood group antigen (HBGA) binding sites, and the blockade epitopes.^14^^,^^15^ HBGAs are glycans expressed on the surface of specific cells and they are determinants of both the ABO and Lewis blood groups. HBGAs are also present in saliva and other bodily secretions. The binding specificity of the VP1 to HBGAs differs between genotypes and results in differences in the susceptibility of individuals to norovirus strains.^16^^,^^17^ Based on the VP1 sequence, the genus *Norovirus* is classified into 10 genogroups (GI-GX) and is further subdivided into 49 genotypes.^18^ Most reported human infections are caused by genogroup GII viruses, from which the GII.4 genotype is the most prevalent.^19^^,^^20^ Antigenic evolution has been related to the GII.4 dominance, for which antigenically distinct variants arise and replace previously established variants.^14^^,^^21^ However, the GII.4 Sydney 2012 variant has been dominant for more than a decade.^22^ In contrast to other genotypes, blockade epitopes (A-I) are well described for GII.4 viruses.^14^^,^^23^

Given that early mucosal immunoglobulin A (IgA) and binding-blocking IgA and IgG anti-norovirus antibodies are the most important correlates of protection,^24^^,^^25^ Ig preparations are expected to contain blocking/neutralizing antibodies against circulating noroviruses, and consequently resolve the infection in patients. Ig preparations are purified antibodies (>98% IgG) obtained from plasma of healthy donors, and they can be administered directly into the duodenum via a nasoduodenal tube to prevent degradation by gastric acid.^7^ Although, the presence of neutralizing antibodies against a common circulating strain has been detected,^5^ the extent of Ig preparations to block/neutralize patient-derived noroviruses has not been tested yet.

Whereas there is evidence of norovirus transmission from immunocompromised individuals to other patients in a nosocomial setting, this has only been shown at early stages of infection.^26^ Recently, it has been shown that some of the late-stage chronic viruses (after several months or years) can still replicate in *in vitro* and *in vivo* culture systems.^27^^,^^28^ However, it is unknown whether these viruses are still transmissible from person-to-person.

The effects of the intra-host quasispecies on HBGA-binding, antigenicity, and the success of Ig therapy, are still poorly understood. Here, we determined the norovirus intra-host evolutionary patterns in chronically infected immunocompromised patients, its relationship with antigenic variation, its consequences for Ig treatment, and the virus potential of transmission between immunocompromised hosts.

## Methods

### Ethics

Written informed consent was obtained from the patients. The study was approved by the Erasmus MC ethical committee under MEC-2015-025 for patients P1–P16.^2^ The study for patients P17–P20 was approved under MEC-2018-1307.

### Patient cohort and sample origin

**Fig. 1:**
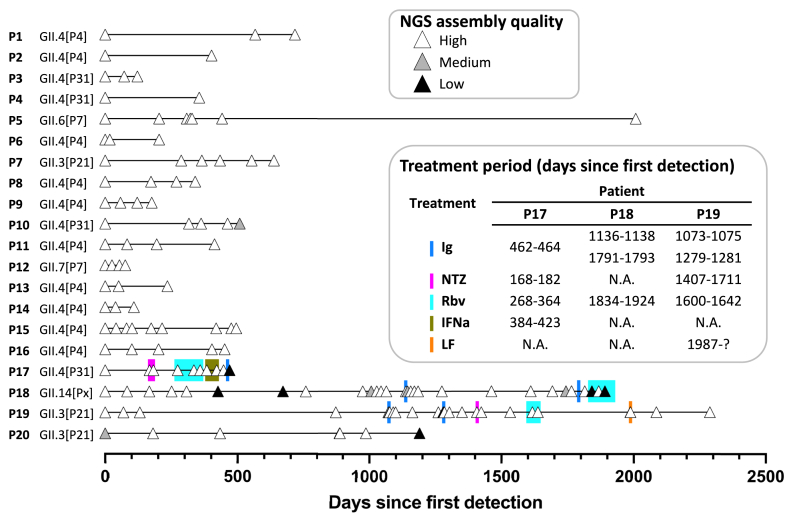
**Timeline of norovirus infections in immunocompromised patients**. Each triangle represents a time point of sampling. Patients P17, P18 and P19 received antiviral treatments against norovirus. The quality of the NGS results was defined as “High” if at least 95% of the norovirus genome had a coverage ≥100×; “Medium” if at least 95% of the genome had a coverage ≥5×; and “Low” if neither of these criteria was met.

A total of 20 immunocompromised patients (P1–P20) chronically infected with norovirus genotypes GII.4 (n = 14), GII.3 (n = 3), GII.6 (n = 1), GII.7 (n = 1) or GII.14 (n = 1) (Fig. 1 and Supplementary Table S1) were included in this study. Seventy-five stool samples from 17 patients (P1–P17) were collected in two previous studies.^2^^,^^5^ Additionally, 65 norovirus positive stool samples were retrospectively selected from the Erasmus University Medical Center biobank and further used for sequencing and cloning. From these samples, 63 were longitudinal samples from three chronically norovirus-infected patients (P18–P20) and two were additional samples from patient-P5 (days 321 and 2008). Stool samples, stored at −80 °C, were used as initial material for both next-generation sequencing (NGS) and cloning of the VP1 gene. In the case of the previously sequenced samples, NGS data was reanalyzed for detection of single nucleotide variants (SNVs). To our knowledge, this study comprises the largest collection of sequenced norovirus samples from chronically infected immunocompromised individuals published to date.

Patients P17, P18 and P19 received multiple off-label treatments throughout the infection, including intra-duodenal delivery of commercial Ig preparations. As described previously, Ig treatment on patient-P17 eliminated symptoms and viral RNA (vRNA) load in feces.^5^ The antiviral treatments of patients P18 and P19 include two courses of Ig treatment each, which did not reduce norovirus infection-related symptoms or vRNA loads in feces. Antiviral treatments were administered as described before.^5^ There is no information regarding the use of antiviral treatments for the other patients during the infection. It should be noted that P17–P20 were patients with genetic disorders (CVID and agammaglobulinemia) characterized by low or absent antibody production, and as such, they received intravenous immunoglobulin (IVIg) treatment multiple times per year. Patients P19 and P20 are siblings sharing the same household. P19 has been infected since 2015, and P20 since 2018, few months after birth. At the time of preparation of this manuscript, patients P18–P20 still exhibit signs of gastroenteritis and remained positive for norovirus vRNA. Confirmed negative norovirus samples were only reported for patients P5 and P17, on days 2547 and 492, respectively.

### Sample processing and genome sequencing

Fecal samples were used as starting material for RNA extraction and generating paired-end libraries. The libraries were enriched for gastrointestinal-disease-causing viruses using an *in-house* set of probes, called Gastrocap. The generated reads, as well as previously generated sequencing files, were used for *de novo* assembly, SNV analysis and haplotype reconstruction. For further global analyses of the SNV data, we excluded samples where quasispecies diversity might not solely result from intrinsic intra-host evolution of the virus (e.g. re- or co-infections and samples after intra-duodenal Ig treatment). Detailed methods for sample processing, sequencing and analysis are described in the Supplementary Methods. Throughout the manuscript, we distinguished between SNV and intra-sample SNV (iSNV). A SNV represents the frequency of reads covering a position that contains a specific mutation compared to the initial (day 0) patient-derived consensus sequence. In contrast, an iSNV is obtained when the consensus sequence of the sample itself is used as reference. Therefore, a SNV can reach a value up to 100%, whereas iSNVs will reach a maximum value of 50%.

The consistency of our Gastrocap method in detecting SNVs across different runs was assessed using three samples from patient P19, showing high correlation between replicates (P-values <0.0001, R^2^ >0.93) (Supplementary Fig. S1). Additionally, we compared the correlation between our new Gastrocap method and the previously used SureSelect capture method (used for patients P1–P16)^2^ for detecting SNVs in five samples from patient P5, showing a high correlation between the two methods (P-values <0.0001, R^2^ >0.91) (Supplementary Fig. S1). These results confirm that the Gastrocap method produces results comparable to those obtained from previously sequenced samples.

### Tree-dimensional (3D) mapping of amino-acid (AA) residues in P-dimer structures

Relevant AA positions were mapped onto the P-dimer 3D structures using PyMOL. The SWISS-MODEL server (https://swissmodel.expasy.org/interactive) was used for homologous modeling of the norovirus P-dimers derived from patient samples corresponding to the AA consensus sequences at day 0. For patients P1, P9 and P17 P-dimer structures were generated by using the P-domain of a GII.4 (PDB number: 5J35). The template for patient-P5 was the Saitama/T87 P-dimer (PDB: 5F4J). VA207 P-dimer (PDB: 3PUM) was used as the template for patients P12 and P18. For GII.4 sequences, blockade epitope (A-H) positions were inferred from Tohma et al. 2021.^15^

### Cloning and expression of RLuc-VP1 fusion proteins

Stool or RNA samples were used for cDNA synthesis and cloning of several complete VP1 genes per sample into the pcDNA3.1-RL-GW plasmid.^29^ VP1 was amplified using PfuUltra II Fusion HS (Agilent Technologies) and cloned using XhoI and XbaI restriction sites. The insert was confirmed by Sanger sequencing. These clonal VP1 sequences were also used for further phylogenetic analysis. The VP1 sequences or their expressed proteins were named as: patient ID_day since first sampling_clone number. For example, “P17-d384_1” refers to a protein obtained from patient-P17 on day 384, clone number 1. A “c” in the last digit stands for the consensus sequence of the sample.

We were unable to clone the VP1 gene for some samples in this study. Therefore, VP1 consensus sequences for patients P1 (P1-d0_c), P18 (P18-d1138, P18-d1273_c and P18-d1815_c) and P19 (P19-d0_c and P19-d874_c) were human codon-optimized and chemically synthetized (Integrated DNA technologies, Inc). The pcDNA3.1-RL-GW plasmid containing the VP1 from a Sydney 2012 strain was previously described.^29^ Other genotype control VP1s were also synthesized and cloned: Hu/NL/2011/GII.4NewOrleans/Rotterdam/11001 (GII.4 NO 2009, OR775692); GII/Hu/NL/2014/GII.21/Groningen (GII.3 Gro_2014, LN854569); calicivirus strain MX (GII.3 MX_2005, U22498); Hu/GII.6/HS245/2010/USA (GII.6_2010, KJ407072); Hu/GII.7/NSW743L/2008/AUS (GII.7_2008, GQ849130); and Hu/GII.4/Beijing/55028/2007/CHN (GII.14_2007, GQ856465). To assess the impact of specific mutations in the VP1 on binding and antigenicity, nucleotide substitutions were introduced into pcDNA3.1-RL-GW plasmids using site-directed mutagenesis. DH5α (Invitrogen) and XL-10 (Agilent) *Escherichia coli* strains were used for cloning. HEK293T cells were transfected using the calcium-phosphate method and RLuc-VP1 fusion proteins were harvested as previously described.^30^ RLuc-VP1 lysates were stored at −80 °C. Lysates were thawed and consistently kept on ice during all assays. The expression of RLuc-VP1 fusion proteins was confirmed by western blot using a rabbit polyclonal anti-RLuc antibody (ThermoFisher Scientific) (Supplementary Fig. S2). HEK293T cells (RRID: CVCL_0063) were obtained from ATCC (CRL-3216) and were not further validated.

### Phylogenetic analysis

The complete VP1 nucleotide gene sequence of representative reference sequences for GII.4 (n = 102), GII.3 (n = 59), GII.6 (n = 54), GII.7 (n = 22), and GII.14 (n = 32) genotypes were retrieved from GenBank and aligned with patient-derived consensus and clonal sequences using MAFFT v7.^31^ The consensus sequences from samples with high and medium sequencing data quality were included.

To investigate the evolutionary history of the noroviruses in this study, we used the Bayesian Markov Chain Monte Carlo (MCMC) method implemented in BEAST 1.10.4.^32^ Molecular clock phylogenies for each genotype were estimated using a relaxed uncorrelated lognormal clock model and the SDR06 nucleotide substitution model. MCMC chains were run for 100–200 million generations and registered every 2000 steps. The first 10% of each MCMC run was discarded as burn-in. Log files were analyzed in Tracer v1.7.1 to confirm that effective sample size (ESS) values were >200, confirming convergence of all parameters. The maximum clade credibility tree was constructed using TreeAnnotator v1.10.4.

Maximum-likelihood trees were constructed in IQtree web server,^33^ using the built-in module for the automatic selection of the best substitution model and the ultrafast bootstrap option with 1000 replicates. Generated trees were visualized utilizing iTol v5 (https://itol.embl.de/)^34^ and Figtree v1.4.4 (http://tree.bio.ed.ac.uk/software/figtree/).

### HBGA characterization of pig gastric mucin and saliva samples

Saliva contains similar HBGAs as the intestine. To evaluate the binding patterns of selected capsids to HBGAs, saliva samples from 8 healthy donors were collected and tested for the presence of specific HBGAs by ELISA (Supplementary Fig. S3). First, saliva samples were centrifuged at 10,000 × *g* for 5 min, and the supernatant was boiled at 100 °C for 5 min. Costar 96 well EIA/RIA flat bottom clear plates (Corning) were coated with either pig gastric mucin type III (PGM-III; Sigma) at 100 μg/mL, saliva samples at a 1:200 dilution or BSA at a 100 μg/mL diluted in PBS. Coated plates were blocked with blocking buffer (1% BSA in PBS with Tween 0.05% (PBS-T)) for 30 min at RT, washed with PBS-T and incubated with HBGA-specific antibodies (Supplementary Table S2) diluted in blocking buffer and incubated for 45 min at 4 °C on a shaker. Plates were washed and incubated with rabbit anti-mouse immunoglobulins/HRP (Agilent) at 1:500 dilution in blocking buffer at 4 °C for 45 min. Plates were washed and developed with 3,3′,5,5′ tetramethylbenzidine (TMB) substrate solution (Thermo Scientific). The Horseradish peroxidase (HRP) enzymatic reaction was stopped by adding 50 μL/well of sulfuric acid [0.25 N]. The assay was read in a Tecan Microplate—Infinite 200 Pro reader at 450 nm absorbance. Antibodies used in this study were obtained from various companies (Supplementary Table S2) and were not additionally validated.

### Luciferase immunoprecipitation system (LIPS)-binding assay

In order to assess the VP1 binding capacity to their glycan receptors, we adapted the LIPS assay developed by van Loben et al. 2021 and Tin et al. 2018^29^^,^^30^ to measure the relative binding of different VP1 proteins to HBGA glycans. An expressed RLuc-VP1 protein retains the capacity to bind the HBGAs through the VP1, meanwhile the RLuc produces a luminescent signal.

Flat-bottom white 96-well Maxisorb plates (Thermo-Fisher Scientific) were coated with PGM-III (100 μg/mL in PBS) or saliva samples (1:1000 in PBS) from the 8 healthy donors described above. Plates were then blocked with blocking buffer at 4 °C for 1 h and washed. The relative light units (RLU) signal of each antigen was determined immediately before starting the assay. RLuc-VP1 lysates were diluted to 10^5^ RLU/μL in blocking buffer to normalize the RLU input. 50 μL of RLuc-VP1 diluted antigen was added in duplicates to PGM-III- or saliva-coated wells and incubated for 1 h at 4 °C while rocking. The bound antigen was detected using the *Renilla* Luciferase Assay System (Promega) in a Tecan Microplate—Infinite 200 Pro reader at 1 s integration time.

### LIPS-blocking assay

Antibody-blocking titers of 14 Ig preparations (Supplementary Table S3) were measured using the LIPS-blocking assay with minor modifications.^29^ Flat-bottom white 96-well Maxisorb plates coated with PGM-III were blocked with blocking buffer. RLuc-VP1 lysates were diluted to 4 × 10^4^ RLU/μL in blocking buffer to normalize RLU input. Ig preparations were diluted 4-fold in blocking buffer, starting with a concentration of 2500 μg/mL, and preincubated with RLuc-VP1 antigen (1:1 volume) in a round-bottom 96-well plate for 90 min at 4 °C while rocking. 100 μL of antibody-VP1 mixtures were added in duplicate to PGM-III-coated wells and incubated for 1 h at 4 °C while rocking. Plates were washed and the bound antigen was detected as before. The mean inhibitory concentration (IC_50_) was defined as the Ig dilution at which the RLU value was 50% of the no antibody control. An IC_50_ value of 5000 μg/mL was assigned to samples that did not show at least a 50% reduction in luciferase activity compared to the control. Graphs were generated using GraphPad Prism 9.0, and IC_50_ values were determined using the nonlinear fit—one site logIC50 binding curve analysis (top constraint = 100, bottom constraint = 0).

### Statistical analysis

For the number of cumulative unique emerging SNVs per gene, we assessed the normality of all analyzed groups using the Shapiro–Wilk normality test. However, many of the groups did not accomplished normality, therefore non-parametric tests were used for the analyses. Statistical differences between genomic regions were determined using the non-parametric Friedman test, followed by the post-hoc Dunn's test for pairwise comparisons. Per each genotype, differences in the blocking activities of Ig preparations against RLuc-VP1 proteins were evaluated by using Kruskall-Wallis followed by Dunn's test for pairwise comparisons. All statistical analyses were performed in GraphPad Prism 9.0.

### Role of the funders

The founders had no roles in study design, data collection, data analysis, data interpretation, or writing of this report.

## Results

### Single nucleotide variant (SNV) analysis reveals selective pressure in the P2 domain

To assess whether there are specific regions in the genome that consistently acquire more mutations than others, we analyzed NGS data from 140 samples from 20 chronically infected patients (Fig. 1 and Supplementary Table S4). For each sample, SNVs to its initial consensus sequence (day 0 of the patient) were retrieved using a SNV frequency cutoff of ≥10%. Only samples with high quality sequencing data were included. To determine the intrinsic patterns of within-host evolution in chronic infections, we excluded samples that could confound this analysis. Therefore, samples with potential re-infections, co-infections, or collected after intra-duodenal Ig treatment were excluded from the analysis (see Supplementary Methods).

**Fig. 2:**
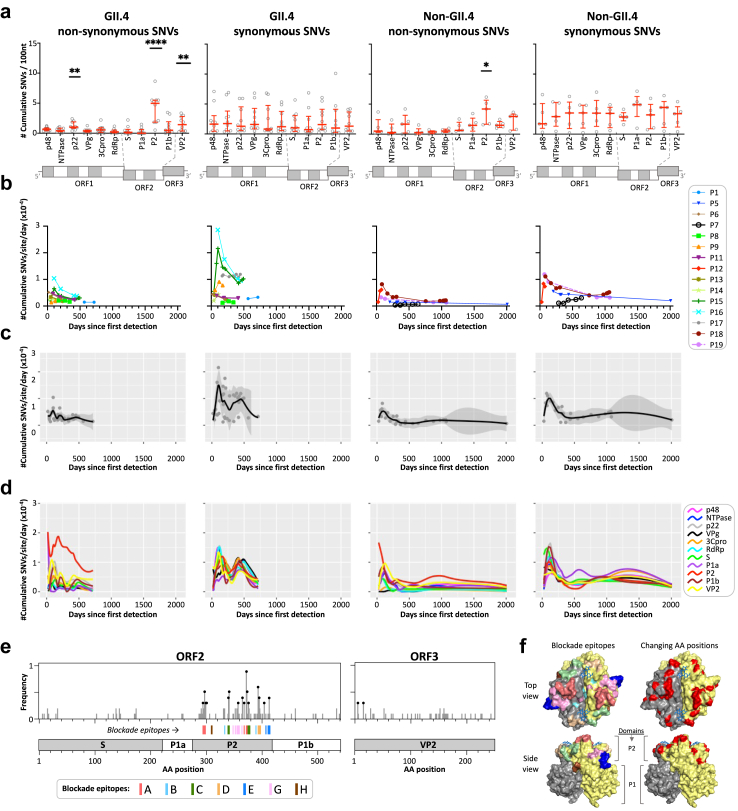
**Patterns of evolution of GII.4 (n = 10) and non-GII.4 (n = 5) noroviruses in immunocompromised individuals by Single nucleotide variant (SNV) analysis**. (a) The number of the cumulative unique emerging SNVs (frequency ≥10%) per gene or protein domains by genotype (GII.4 and non-GII.4) and type of mutation (non-synonymous and synonymous). SNVs that were present at first day of collection (day 0) were not count for calculations. The median and the interquartile range are shown in red. P values were determined by comparing each gene to the correspondent RdRp. ∗P < 0.05, ∗∗P < 0.01 and ∗∗∗∗P < 0.0001. (b) Rate of mutations over time (cumulative unique emerging SNVs per site per day) in the genome of each individual patient by genotype and type of mutation. (c) Rate of mutations over time in the genome of aggregated patient data by genotype and type of mutation. The curves show LOESS fits, and shaded areas show 95% confidence intervals, as implemented in the geom_smooth function in ggplot2 R package. (d) Rate of mutations over time per gene of aggregated patient data by genotype and type of mutation. Only LOESS curves are shown per gene. (e) Analysis of the amino-acid (AA) position hotspots in GII.4 ORF2 and ORF3 genes. The x-axis indicates the AA positions of the protein relative to the GII.4 Sy 2012 reference (JX459908), while the y-axis indicates the frequency of patients (n = 10) in which the initial AA has changed at the consensus level (SNV frequency >50%). Positions with a frequency ≥0.3 (at least 3 patients) are shown as black circles. (f) GII.4 P-dimer structure (PDB: 5J35) showing both the blockade epitopes and the AA position hotspots of mutation. Blockade epitopes are colored based on panel e.

The number of non-synonymous or synonymous cumulative unique emerging SNVs (mutations that newly arose after the first day of sampling) for each patient was calculated and normalized by the length of the gene or domain (Fig. 2a). The number of cumulative unique emerging synonymous SNVs was similar across all genes for GII.4 (n = 10) and non-GII.4 (n = 5) viruses. The P2 domain showed the highest number of cumulative non-synonymous SNVs (Dunn's multiple comparisons test against the RdRp, P-value <0.0001), with a median between 3.5 and 31.6-fold higher compared to the other genomic regions for GII.4 viruses and between 1.4 and 15.4-fold higher (P-value = 0.031) for non-GII.4 viruses. The p22 and VP2 genes of GII.4s also showed a significantly higher number of cumulative non-synonymous SNVs compared to the RdRp (P-value = 0.0035 for both), which was not observed for non-GII.4s, probably due to the smaller sample size.

To investigate the dynamics of mutations throughout the course of infection, we calculated the mutation rate as the number of cumulative unique emerging SNVs per site per day for each patient (Fig. 2b). We observed variable mutation rates between and within patients, with up to 4.5-fold difference between time points from the same host (patient-P18), regardless of the type of SNV or genotype. To visualize the overall patterns of mutation rates, we aggregated the data by genotype (Fig. 2c). Interestingly, a higher mutation rate was observed soon after the day of first detection for both genotype groups. Analysis per gene (Fig. 2d) showed that, as expected, the P2 domain has a higher mutation rate at the non-synonymous level compared to other genes. Remarkably, GII.4 viruses seem to sustain a higher accumulation of non-synonymous mutations in the P2 domain over time compared to non-GII.4 viruses. To exclude the possibility that these analyses were convoluted by cases where there was a considerable gap between the first day of sampling and the actual onset of infection, we re-analyzed the data, including only those patients with evidence of sampling shortly after infection onset (Supplementary Methods and Supplementary Table S5). In this subset of samples (P1, P6, P11, P13 and P14), all of which were GII.4s, similar evolutionary patterns were observed, although the differences were less pronounced compared to the full dataset (Supplementary Fig. S4a–d). The P2 domain showed the highest accumulation of non-synonymous mutations (P-value = 0.0085), while no significant differences were observed for other genes (Supplementary Fig. S4a). The mutation rate showed up to a 2-fold difference between time points from the same host (patient-P11) (Supplementary Fig. S4b), This may be explained by a rapid initial diversification of the virus, after which the fittest viral subpopulations remain relatively stable over time. Finally, the P2 domain showed a higher mutation rate at the non-synonymous level compared to other genes (Supplementary Fig. S4d).

Next, we determined the AA position hotspots subject to higher evolutionary pressure in ORF2 and ORF3 for GII.4 viruses. A total of 18 AA positions changed at the consensus level in at least 30% (3/10) of the cases, reaching up to 90% for residue 372 of the ORF2, which is part of blockade epitope A (Fig. 2e and Supplementary Table S6). In fact, 13 of these AA positions were surface residues in known blockade epitopes within the P2 domain (Fig. 2e and f), with epitopes A, C and D showing 6/8, 3/7 and 2/5 position hotspots, respectively. Interestingly, no AA substitutions were found in the conserved protective epitopes B, H, and I^23^ in any of these patients (Supplementary Table S6). This analysis was not extended to other genotypes due to the limited number of patients.

Additionally, haplotypes reconstructed from these samples were used to detect signals of positive (diversifying) and negative (purifying) selection in the codons of the GII.4 VP1 for each individual. Only haplotypes with a frequency ≥1% were included (Supplementary Methods). A total of 8 codons, 7 of which are in the P2 domain, were identified as being under diversifying selection in at least two patients (Supplementary Table S7). Codon positions 299, 341, 372 and 413 coincided with those that changed most frequently at the consensus level (Supplementary Table S6). Purifying selection was observed for 29 codons throughout the VP1, including position 310 within epitope H (Supplementary Table S7). This suggests a fundamental role for some of these residues in viral fitness during chronic infections.

### Norovirus quasispecies cluster into multiple lineages during chronic infections

**Fig. 3:**
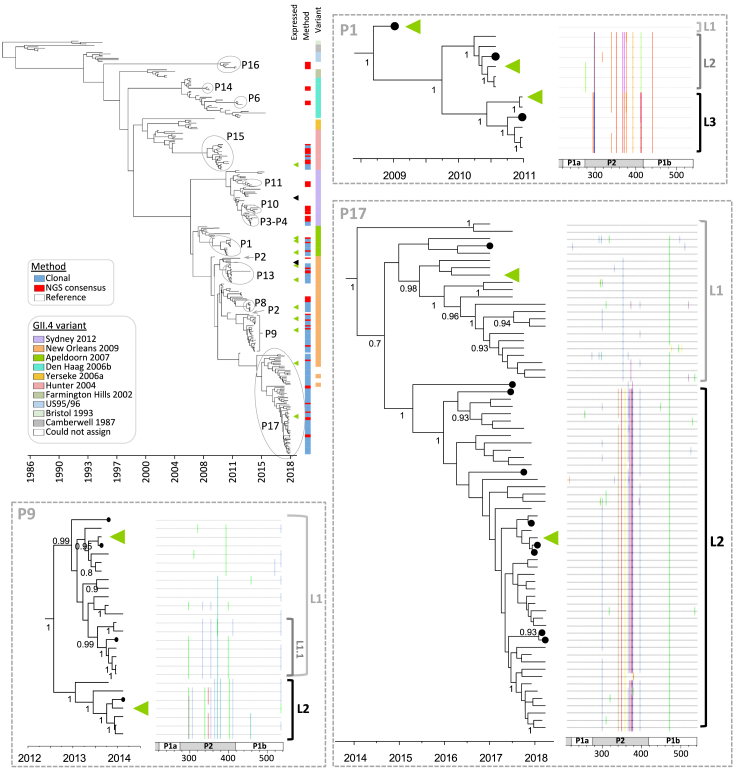
**Time-scaled phylogenetic tree for GII.4 noroviruses**. The tree was constructed from clonal and NGS consensus VP1 nucleotide sequences of norovirus GII.4 from 14 chronically infected patients, along with reference sequences. Patient-derived sequences are indicated in the main tree. Green triangles indicate representative patient-derived VP1 sequences, while black triangles indicate reference VP1 sequences that were further expressed and characterized. Specific subtrees show the intra-host norovirus diversity within patients P1, P9, and P17. Posterior values above 0.7 are shown. Consensus sequences are shown as black circles. Matched highlighter plots show AA substitutions in the P-domain relative to the top sequence in the phylogeny. Trees for the non-GII.4 viruses are shown in Supplementary Fig. S5.

To investigate quasispecies heterogeneity and its temporal changes, we cloned and sequenced the VP1 gene from longitudinal samples of 10 patients (P1, P3, P5, P8, P9, P12, P13, P15, P17, and P18). For 5 patients (P1, P5, P9, P12 and P17) we obtained clones of at least 2 samples representing norovirus diversity over time. Phylogenetic trees were inferred using 3–17 clonal VP1 sequences for each time point per patient, all available NGS consensus sequences and references from GenBank. The main trees showed that in some cases viruses derived from chronic infections (e.g., P16, P15, P1, P13, P9, P17) are highly divergent from commonly circulating strains (Fig. 3 and Supplementary Fig. S5). For both GII.4 and non-GII.4 viruses, between 2 and 3 clearly defined major lineages, that were robustly supported by posterior values ≥0.7, could be distinguished. To extend this analysis to samples with few or no clones obtained, we reconstructed haplotypes of the VP1 from NGS data for all samples. The presence of major lineages in these samples was confirmed by multidimensional scaling analysis based on amino-acid distances of both clones and inferred haplotypes (Supplementary Fig. S6). Lineages were defined based on tree topology and the presence of linked AA substitutions with potential impact on the receptor binding site or antigenicity. A sub-lineage was defined as a sub-group of clones clustering together within a lineage and exhibiting additional AA changes common to this sub-group. A (sub-)lineage-defining mutation was established as a non-synonymous mutation present in >80% of sequences belonging to the lineage and <20% in any other lineage. The majority of intra-host lineage-defining mutations were in the P2 domain (Fig. 3 and Supplementary Fig. S5).

**Fig. 4:**
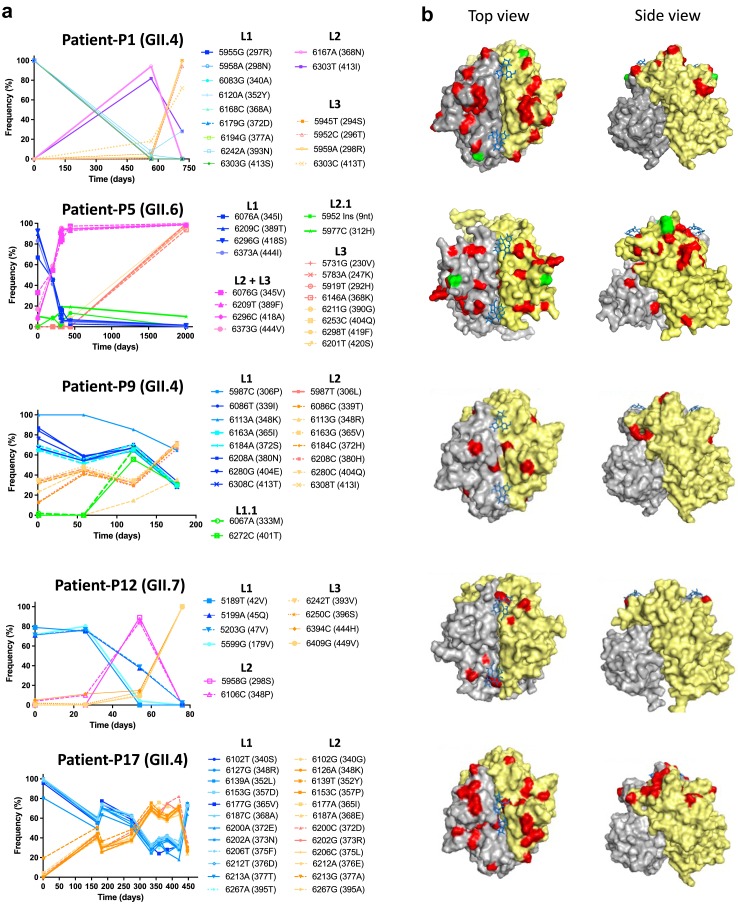
**Intra-host lineage dynamics based on the frequency of lineage-defining mutations over time**. (a) Frequency of lineage-defining mutations to estimate lineage dynamics. Lineage-defining mutations of a (sub-)lineage were determined based on their prevalence in clonal sequences of such lineage. The frequency of each mutation was determined by SNV analysis, where only positions with a coverage ≥100×, Phred score ≥30, a frequency ≥1%, and at least three reads containing the SNV at the specific nucleotide position were considered. VP1 residue positions are given relative to the initial (day 0) VP1 consensus AA sequence derived from each patient. For all GII.4 sequences, the AA positions align with those of the Sydney 2012 reference sequence (JX459908). For patient P5, lineage-defining mutations of L2 are included within L3. (b) Norovirus P-dimer structure of the intra-host lineages. Residues shown in red and green denote within-host AA changes and indels between lineages of the same patient, respectively. HBGA glycans are shown in blue.

The frequencies of lineage-defining mutations in NGS data were used to estimate the abundance of each intra-host lineage during infection (Fig. 4a). Two clear patterns were observed: (i) Co-circulation, where one of the lineages is more prevalent at the day of first detection, and as a new viral lineage emerges, both populations are maintained over time. This was observed for patients P9 and P17; (ii) Lineage replacement, where an initial lineage dominates the viral quasispecies population and is replaced by one or more new lineages. This was observed for patients P1, P5, and P12, with lineage replacements occurring twice for patients P1 and P12. Hereafter, the first lineage detected in a patient is referred as lineage-L1, the second as lineage-L2, and so on. Due to the limited number of samples that could be cloned, these analyses were not performed for other patients. However, similar dynamics of the lineages were observed when haplotype sequences were used to estimate the abundance of each intra-host lineage (Supplementary Fig. S7).

To visualize the spatial distribution of the AA changes, corresponding to the lineage-defining mutations, we mapped them onto P-dimer structures. For all cases, the majority of changes were located at exposed residues within the P2 domain, in close proximity to the HBGA binding pocket (Fig. 4b). Specifically, 19 out of 27 (70%) AA positions where GII.4 intra-host lineages (P1, P9 and P17) differed from each other, were located within the blockade epitopes A-I (Supplementary Fig. S8).

### Norovirus intra-host differences in binding to HBGAs

To investigate the phenotypic differences among inter- and intra-host virus populations, we expressed representative patient-derived VP1s fused with the *Renilla* Luciferase (RLuc), called RLuc-VP1 proteins.^29^ A total of 25 VP1s representing viruses from 11 patients were selected (green arrows in Fig. 3 and Supplementary Fig. S5) based on if they contained all signature mutations of a lineage or as consensus sequences from samples obtained after Ig treatment. Additionally, seven genotype controls were included. The VP1 sequences or their expressed proteins were named according to the format: patient ID_day since first sampling_clone number. A ‘c' in the last digit denotes the consensus sequence of the sample.

**Fig. 5:**
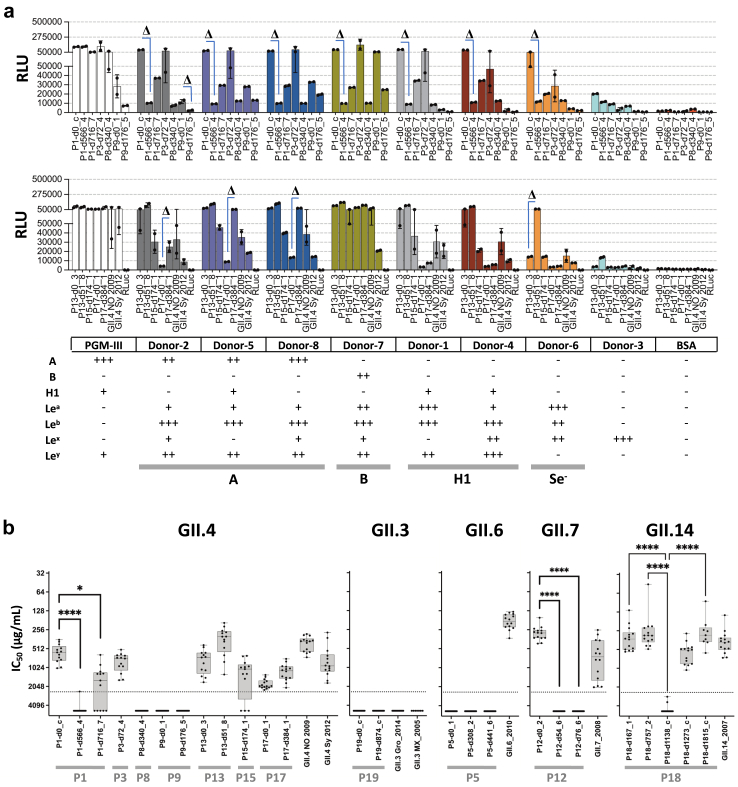
**Phenotypic characterization of patient-derived norovirus capsids**. (a) Binding of GII.4-derived RLuc-VP1 fusion proteins to PGM-III and saliva containing HBGA. The luciferase activity of each RLuc-VP1 protein was adjusted to 5 × 10^6^ RLU/well before performing the binding assay. Plots are grouped by genotype and HBGA type (bottom table). The average RLU signal ± standard deviation of two independent experiments are shown. The presence of specific HBGAs in the 8 saliva samples (D1-D8) was tested by ELISA (Supplementary Fig. S3). Differences in binding of >4-fold (Δ) between intra-host-derived proteins are indicated. The HBGA-binding profile for non-GII.4-derived RLuc-VP1 proteins is shown in Supplementary Fig. S9. (b) Blocking activities of Ig preparations against different RLuc-VP1 antigens. Fourteen commercial Ig preparations were tested for their HBGA binding-blocking activity of patient and genotype control RLuc-VP1 proteins to PGM, using dilutions of the Ig preparations where the starting concentration was 2500 μg/mL. Plots are grouped by genotype, and each dot represents the titer of an Ig preparation versus an antigen. Ig preparations with non-blocking activity detected are shown as IC_50_ = 5000 μg/mL. Only significant differences between intra-host-derived proteins are shown. ∗P < 0.05 and ∗∗∗∗P < 0.0001.

First, we determined the relative binding capacity of the VP1s to HBGAs in pig gastric mucin type-III (PGM-III) and a panel of 8 saliva samples from healthy donors with various HBGA profiles (bottom panel in Fig. 5a). The saliva samples included individuals with HBGAs A, B, H1, and a non-secretor (Se^−^), representing the majority of the population. A minimum of 4-fold difference in the luciferase signal was considered a substantial difference. Most VP1s bound PGM-III at similar levels when viruses derived from the same patient were compared (Fig. 5a and Supplementary Fig. S9). Overall, salivary HBGA-binding profiles were heterogeneous both within and between hosts. Within-patient differences were observed in 6 out of the 8 patients from which at least two VP1s were expressed (P1, P5, P9, P12, P13, P17, P18 and P19), reaching a 55.9-fold difference between P12-d0_1 (lineage-L1) and P12-d54_6 (lineage-L2) proteins against saliva type B (Fig. 5a and Supplementary Fig. S9). Interestingly, for patient-13, P13-d51_8 showed >4-fold higher binding to saliva of donor-6 compared to P13-d0_3, despite having only one AA difference (P396H) in epitope D (Supplementary Fig. S8). In general, all GII.3 VP1s showed low binding to all saliva samples. For patient-P18 (GII.14), P18-d1138_c, the consensus obtained after the first Ig treatment, showed up to >30-fold lower binding to salivas than other P18-derived proteins. Hence, the binding pattern to salivary HBGAs can vary between intra-host viruses, indicating that their AA differences can affect binding specificity and/or affinity.

### Antigenic differences between- and within-host viruses

To characterize the antigenic variation of the patient-derived viruses and to determine the variability of the HBGA-binding-blocking capacity of different Ig preparations, we evaluated the blocking activity of 14 commercial Ig preparations (Supplementary Table S3) against all RLuc-VP1s. Ig-3 was a matching batch with the Ig preparation used for the second treatment of patient-P19. The 50% inhibitory concentration (IC_50_) was determined for each antigen-Ig combination, with lower values indicating a higher blocking capacity. In general, all genotype controls (except for GII.3s) were blocked by all Ig preparations, whereas 12 out of 25 (48%) patient-derived proteins were widely resistant, with IC_50_ values outside of the detection limit (Fig. 5b). In general, there was high variability in the blocking capacity of the Ig preparations to different antigens, even within a single genotype (Fig. 5b), but minimal differences in the blocking patterns between Ig preparations. Hierarchical clustering based on IC_50_ values showed 4 Ig groups with bootstrap values >70 (Supplementary Fig. S10). These groups did not correspond with a specific brand or production date.

At the intra-host level, VP1s derived from 3 patients (P1, P12 and P18), out of the 8 for which at least two VP1s were expressed, showed clear differences in their HBGA-binding-blocking sensitivity (Fig. 5b). For viruses derived from patients P1 (GII.4) and P12 (GII.7), VP1s representing the early lineages (day 0) were significantly more blocked than later ones (Kruskal–Wallis test, P-value <0.0001) (Fig. 5b). Between 4 and 15 AAs in the P2 domain differentiate those early and late viruses (Supplementary Fig. S8), and likely explain the observed antigenic variation. For patient-P18 (GII.14), all proteins showed similar blocking sensitivity levels to Ig preparations, except for P18-d1138_c. This protein contains three unique AA residues (310T, 343I, and 369Q) that were absent in the other expressed proteins derived from this patient and the GII.14 control (Supplementary Fig. S8), suggesting a role in conferring resistance to Ig treatment. Notably, P18-d1815_c, the VP1 consensus sequence of the virus 22 days after the second Ig treatment, was sensitive to blocking antibodies. In conclusion, we observed significant differences between- and within-host viruses in their HBGA-binding-blocking sensitivity towards Ig preparations, but limited variation between Ig preparations.

### Impact of antiviral and Ig treatments on norovirus within-host populations

In principle, antivirals can exert evolutionary pressure on intra-host virus populations. Depending on the susceptibility of the viral population to such antivirals, the outcome can vary from no changes in the intra-host virus populations to complete clearance of the infection. To evaluate the effects of different off-label treatments on viral populations, we assessed SNV data from the three patients (P17–P19) who received such treatments.

**Fig. 6:**
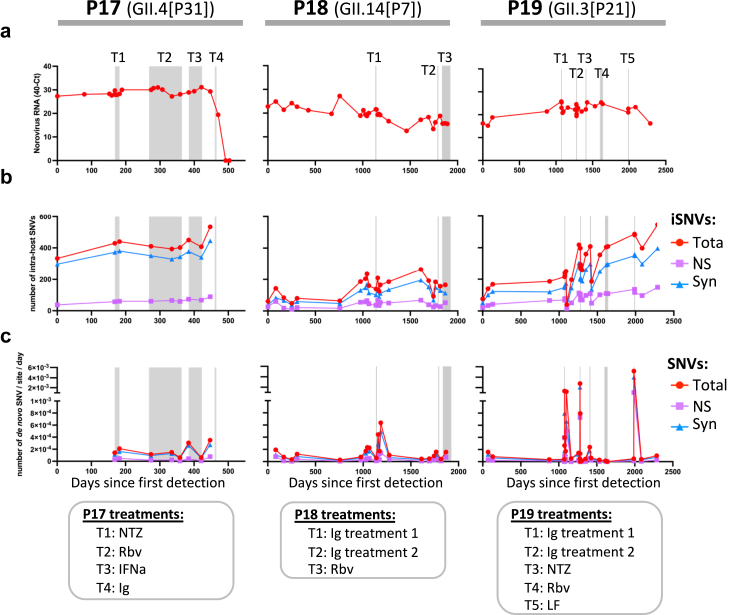
**Effects of antiviral treatments on norovirus intra-host populations**. (a) Norovirus RNA levels in feces of immunocompromised individuals under antiviral treatment (n = 3). (b) Number of intra-sample SNVs (iSNVs: variants relative to the consensus sequence of the sample) over time. (c) Rate of *de novo* SNVs emerging over time. For panels b and c, only SNVs present at ≥10% of the reads were considered for the analysis.

For patient-P17, NTZ, Rbv and IFNa did not have a detectable impact on vRNA levels, while Ig treatment cleared the infection.^5^ For patients P18 and P19, none of the treatments, including Ig treatments, decreased vRNA (Fig. 6a). Next, the within-host diversity over time was assessed by plotting the number of iSNVs. It is expected that a treatment that creates a bottleneck in the virus population will result in a decrease in the number of iSNVs. This was clearly observed for patient-P19 on day 1099, 26 days after the first Ig treatment, where the number of iSNV decreased from >200 to 40 (of which 15 were non-synonymous), suggesting a strong bottleneck in the viral population (Fig. 6b). A third aspect evaluated was the rate of *de novo* mutations (number of *de novo* SNVs per site per day). Presumably, an evolutionary bottleneck in the population will give rise to novel mutations causing resistance. We observed this for patient-P18 after the first Ig treatment and for P19 after each Ig treatment (Fig. 6c). To explore potential mutations that could cause resistance to antiviral treatments, we plotted the frequency of non-synonymous SNVs that became dominant (≥50%) soon after or during each of the treatments (Supplementary Fig. S11). NTZ and IFNa treatments (P17 and P19) seem to have minimal impact on the emergence of specific non-synonymous mutations, given that AAs that became prevalent were already present at considerable levels (>20%) before each treatment. Rbv treatment was associated with the emergence of several AA substitutions for patients P17 and P18, from which 3 were found in the RdRp of each patient, but not for patient-P19. Ig treatment was associated with emergence of mutations mainly in the capsid, especially in the P2 domain. For patient-P18, two substitutions in the P2 domain became transiently dominant immediately after the first Ig treatment (N343I and S310T), and 3 (N285S, Q328R and H378R) after the second Ig treatment. For patient-P19, ten substitutions in the VP1, from which 9 were located in the P2 domain, clearly increased after the first Ig treatment, shifting from <10% to 68–100% frequency. After the second Ig treatment, three AA substitutions reached consensus; however, they were present at >20% frequency before treatment. Following the lactoferrin (LF) treatment, four AA substitutions in the P2 domain and five in non-structural proteins (p48 and VPg) reached consensus. Notably, the T291A substitution in the P2 domain became dominant after both the first Ig and the LF treatments. It has been suggested that LF affects norovirus replication by activating the innate immune system and preventing the virus from binding to its glycan receptors through attachment to the VP1.^35^^,^^36^ Further research is needed to assess the role of these mutations in overcoming these antivirals.

### Single AA substitution can cause resistance to the blocking activity of Ig preparations

**Fig. 7:**
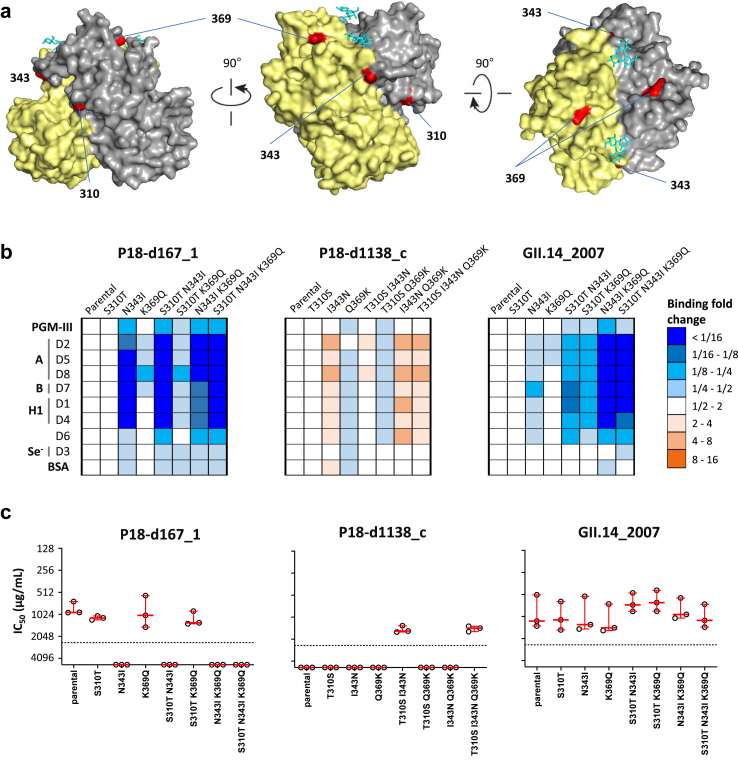
**HBGA-binding and antigenic activities of P18-derived norovirus (GII.14) VP1 mutants**. (a) P-dimer crystal structure of the P18_d0 consensus showing the localization of the three residues potentially involved in the resistance to Ig treatment. (b) Fold-change in the binding of RLuc-VP1 proteins to PGM-III and saliva samples containing HBGAs. Each graph shows the fold change in RLUs of the mutant proteins relative to their parental protein backbone. Plotted values are the average of two independent experiments. (c) Blocking activity of three Ig preparations (Ig-3, Ig-14, and Ig-15) against the RLuc-VP1 proteins.

Considering that Ig treatments failed for patient-P18, and that the P18-d1138_c protein (consensus after first Ig treatment) exhibits distinct HBGA-binding and antigenic profiles compared to the other expressed VP1s from the patient (Fig. 5b and Supplementary Fig. S9), we evaluated whether one or more of the three unique AA substitutions in the P2 domain of P18-d1138_c (Supplementary Fig. S8) are responsible for the observed phenotype. The three residues (AA positions: 310, 343, and 369) are exposed at the surface of the P-domain (Fig. 7a). To assess their individual or combined contributions, we generated single, double, and triple mutants utilizing three distinct protein backbones: P18-d167_1, which represents an Ig-sensitive early clone; P18-d1138_c, the Ig-resistant strain; and GII.14_2007, as Ig-sensitive genotype control.

A single AA substitution of asparagine (N) to isoleucine (I) at position 343 (N343I) in both P18-d167_1 and GII.14_2007 VP1s substantially reduced (>4-fold) binding affinity to HBGAs (Fig. 7b). Conversely, the reverse substitution, I343N, had the opposite effect on the P18-d1138_c backbone (Fig. 7b), indicating the importance of this substitution for HBGA binding.

Next, we determined the binding-blocking titers of three Ig preparations against all mutants (Fig. 7c). For P18-d167_1, N343I alone conferred resistance to Ig blocking activity, whereas the reverse substitution (I343N) in the P18-d1138_c resistant backbone did not restore sensitivity to Ig-blocking activity. However, the combination of T310S and I343N substitutions restored the sensitivity to Ig-blocking antibodies. Interestingly, all GII.14_2007 VP1 mutants maintained their sensitivity to Ig-blocking antibodies, indicating that equivalent AA substitutions can have distinct antigenic effects in different capsids, even among closely related proteins (i.e., the same genotype).

### Transmission of late-stage viruses between immunocompromised individuals

**Fig. 8:**
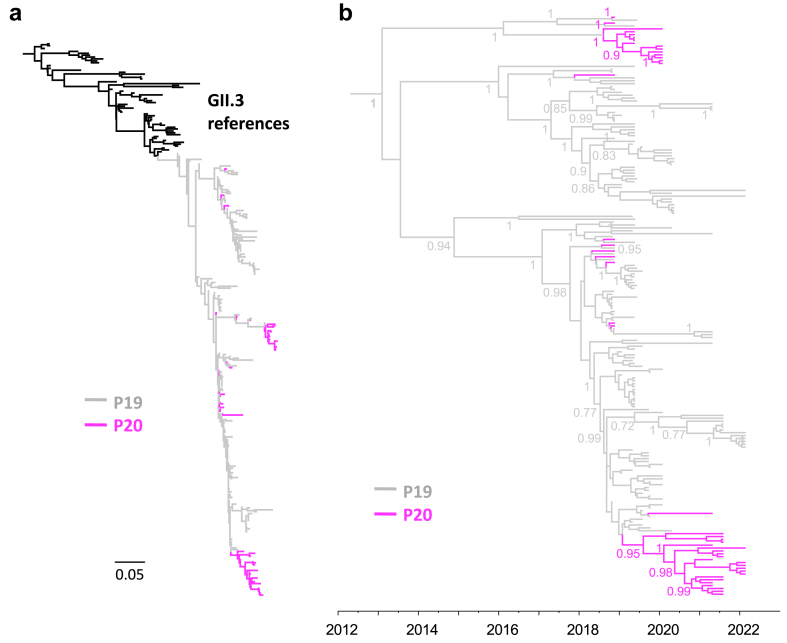
**Phylogenetic evidence of person-to-person transmission between two immunocompromised individuals**. (a) Maximum-Likelihood tree constructed from consensus (n = 34) and clonal (n = 201) sequences of the P-domain (768 nucleotides) derived from P19 and P20, along with GII.3 reference sequences (n = 60) from GenBank. (b) Time-scaled phylogenetic tree of the clonal sequences derived from P19 and P20. Selected posterior values >0.7 are shown.

Given that the VP1 consensus sequences from patients P19 and P20 formed a monophyletic group (Supplementary Fig. S5) and the clear epidemiological link between the two patients, we examined their within-host viral population composition in more detail. We cloned the P-domain from all seven samples of patient P20 and 21 samples of patient P19 starting from day 1077 (a month before patient P20 first tested positive for norovirus). A total of 201 P-domain clonal sequences were obtained. A maximum-likelihood tree based on clonal, consensus and reference sequences showed that all P19–P20 sequences form a single monophyletic group (Fig. 8a), with no evidence of co-infection by a new norovirus strain. A time-scaled phylogenetic tree of the clonal sequences indicated that the initial viral population of patient P20 (P20-d0, November 2018) was composed of diverse sequences distributed throughout the tree (Fig. 8b). This suggests that early in the infection, virus quasispecies were transmitted from P19 to P20, potentially through a single transmission event involving various haplotypes or through multiple transmission events. However, later on, only two main clusters of viruses predominated in the viral population of P20. This was confirmed by a temporal tree of predicted haplotype VP1 sequences (Supplementary Fig. S12). Additionally, Human Astrovirus 1 (HAstV-1) sequences were also assembled from P19 (since day 1090) and P20 (since day 0) samples, showing that these viruses originated from the same source and most likely resulted from transmission between these two patients (Supplementary Fig. S13).

## Discussion

With the progress in healthcare and a rise in possible therapeutics, the life expectancy of immunocompromised patients has dramatically increased in the last decades. Nevertheless, chronic diarrhea is still a major concern for the quality of life of these patients, with norovirus as one of the main causes.^1^^,^^8^^,^^37^ Furthermore, the COVID-19 pandemic has highlighted the potential of such hosts as a reservoir for evolution of novel virus variants.^38^ To gain insights into the common traits of the within-host evolution of norovirus and their effects for treatment in immunocompromised individuals, we analyzed a comprehensive dataset of longitudinal samples and characterized the VP1 of representative viruses. Notably, these intra-host viruses tend to acquire the most non-synonymous changes in the P2 domain, evolving as subpopulations or lineages that frequently exhibit distinct phenotypes. Besides, within-host viral subpopulations, harboring few specific AA residues, appear to be the responsible of Ig treatment failure.

Intra-host viruses frequently display phenotypic differences among each other and to community-circulating viruses. Although several studies have described that noroviruses during chronic infections tend to accumulate a high number of mutations,^2^^,^^11^, ^12^, ^13^^,^^39^ their impact on functionality is rarely further characterized. In this study, we found clear antigenic differences between intra-host viruses for at least three patients. Moreover, several (∼50%) of the patient-derived viruses were broadly resistant to Ig preparations, suggesting that they are antigenically distinct to community-circulating strains. Given that Ig preparations are pools of polyclonal antibodies from many donors, the antigenic differences between lineages were presumably underestimated. Homotypic sera raised against specific intra-host viruses will better elucidate antigenic differences between intra-patient lineages. Similarly, HBGA-binding patterns were heterogeneous, even between VP1s representing intra-patient viruses. Taking all together, we have shown that genetic differences between intra-host strains often result in HBGA-binding specificity and antigenicity changes.

While most data to date suggests that norovirus tends to accumulate most mutations in the P2 domain during chronic infections,^11^, ^12^, ^13^^,^^40^^,^^41^ this has not been statistically tested, nor quantified. In addition, other regions of the genome are often overlooked. Here we have used NGS data to assess the intra-host norovirus mutation patterns along the genome of GII.4 and non-GII.4 viruses. We observed that non-synonymous mutations tend to emerge up to 30 times more often in the P2 domain than in other genomic regions, with GII.4s showing a prolonged higher rate of non-synonymous mutations in the P2 than non-GII.4s, probably due to differences in the structural constraints among genotypes, but more studies are needed to validate this observation. The high acquisition of non-synonymous mutations in the P2 domain, and recent evidence showing that serum antibodies against the VP1 increase over time in chronic infections,^13^ strongly indicate that antibody-mediated immune pressure is the primary evolutionary mechanism for noroviruses within immunocompromised individuals. While antibody-mediated immunity is likely the main driver of intra-host evolution in the P2 domain, other selection mechanisms may contribute to the accumulation of non-synonymous mutations in the VP2 and p22 genes, such as T cell-mediated immunity and adaptation to the host environment. Indeed, both p22 and VP2 proteins have been suggested as regulators of the innate and adaptive immune responses^42^; however, their exact role in sustaining infections in immunocompromised individuals remains unknown. Additionally, for GII.4s, the elevated accumulation of non-synonymous mutations in the P2, p22 and VP2 genes is similar to the evolutionary patterns observed in community-circulating noroviruses, where both the evolutionary rate (substitution/site/year) and the Shannon entropy (degree of information contained in a sequence) were also high for P2, VP2, and p22 genes.^43^^,^^44^ Moreover, most of the AA position hotspots for GII.4 viruses overlap with those that constitute the blockade epitopes (especially A, C and D) which are the main drivers of the antigenic differences between variants.^15^ Given that our analysis excluded samples after intra-duodenal Ig treatment and potential re- or co-infections, we showed that the intrinsic intra-host norovirus evolution in chronic infections resembles that observed at the community level.

Norovirus quasispecies in chronic infections tends to evolve in lineages. These observations are consistent with previous findings in other chronically norovirus-infected patients, showing the presence of one or more viral subpopulation clusters (here referred as lineages) within each individual over the course of infection.^13^^,^^39^^,^^45^ Furthermore, this study revealed two possible modes of evolution during chronic infections: lineage replacement and lineage co-circulation. It has been previously observed that, during co-circulation, lineage(s) can remain relatively stable over time, with minimal accumulation of mutations in each lineage subpopulation.^39^^,^^45^ The factors determining which mode of evolution occurs may include: 1) Compartmentalization, which is supported by varying *in vitro* replication efficiencies in duodenal-versus ileum-derived human intestinal epithelial cells.^46^ Salivary glands are also potential sites of compartmentalized replication, where norovirus and other enteric viruses can replicate.^47^ Remarkably, evidence of differential acquisition of mutations due to compartmentalization has been observed for viruses such as poliovirus, rhinovirus, HIV-1, and SARS-CoV-2^48^, ^49^, ^50^, ^51^, ^52^; 2) patient immune status, where lineage replacement could be favored in individuals who exert relatively high immune pressures; and 3) cooperative interactions among intra-host viral lineages, for example, by playing complementary roles in the mitigation of neutralizing immune responses as suggested for HCV.^53^^,^^54^

The use of Ig preparations in treating chronic norovirus infections has shown varying results, leading to the clearance of infection in some patients,^5^^,^^8^^,^^10^^,^^55^^,^^56^ but not in others.^9^^,^^57^, ^58^, ^59^ The reasons for these differences are not well understood. However, the route of administration (i.e., intravenous versus intra-duodenal), inter-batch variability, or the virus genotype are some of the most likely explanations for these observations. Generally, differences in binding-blocking susceptibility to Ig preparations between VP1 proteins were not only genotype-dependent but also intra-host strain-dependent. Interestingly, for patient-P18-derived noroviruses, the susceptibility to Ig changed by introducing only 1 or 2 AA substitutions, but those same AA changes were not able to alter the susceptibility of the GII.14_2007 genotype control. This asymmetric antigenic effect suggests that epistatic interactions play a fundamental role in norovirus antigenic evolution. Similar observations have been reported for HIV-1, influenza, and SARS-CoV-2.^60^, ^61^, ^62^ Interestingly, none of the AA substitutions that emerged after each of the Ig treatments on patient-P18 were present in other GII.14 sequences in GenBank, suggesting that those AAs have a relatively high fitness cost. Thus, under a strong evolutionary pressure, as the one caused by Ig treatment, viral subpopulations harboring resistant AA residues would transiently take over and decrease when the pressure stops. In light of the high viral diversity found in chronic norovirus infections, it is reasonable to think that the presence of intra-host subpopulations or lineages antigenically distant to community-circulating strains are the major contributors to Ig treatment failure. As the infection progresses, there is an increased likelihood of emergence of viral subpopulations resistant to Ig treatment. Therefore, timely administration of Ig might enhance its effectiveness. In order to improve treatment outcome, future therapeutic strategies could include the use of broadly neutralizing antibodies, nanobodies^63^^,^^64^ or tailor-made polyclonal antibodies covering the within-patient norovirus antigenic diversity. The finding that some protective epitopes are well conserved during chronic infections gives confidence that broadly neutralizing antibodies are possible, even for these divergent and heterogenous viruses. Alternatively, the expression of representative patient-derived VP1s using a rapid method such as the LIPS assay^29^ can be used for pre-screening of blocking activity against specific viruses infecting the patient. This approach can aid in selecting an appropriate batch of Ig preparation or anti-norovirus convalescent sera for treatment.

Some characteristics of the norovirus evolution in immunocompromised patients resemble those observed in community-circulating noroviruses, including the emergence of antigenically distinct viruses. What it is more, chronic infections may explain the emergence of new variants under specific contexts. Mathematical models suggest that immunocompromised individuals may significantly impact antigenic evolution of viruses like SARS-CoV-2 when epistasis (interaction between residues) is involved.^65^ In addition, person-to-person transmission of late-stage viruses was observed between immunocompromised individuals. Although this raises concerns on potential transmissions between these patients in nosocomial settings, there is no indication that these viruses have infected immunocompetent individuals and have caused large outbreaks. Other potential sources of novel noroviruses could include zoonosis^66^ or immunocompetent children.^67^ Ultimately, the role of immunocompromised individuals in the emergence of novel noroviruses and other RNA viruses is yet to be determined.

In summary, this study demonstrates that intra-host norovirus populations in immunocompromised individuals are composed of diverse subpopulations with varying antigenic and binding specificities. This knowledge can help to improve therapeutic strategies for chronic infections.

### Limitations of the study

The small number of patients infected with non-GII.4 viruses did not allow us to determine the details of evolutionary patterns and mutational hotspots for other genotypes. Combining datasets from multiple institutions could provide greater insights into the evolution of these other genotypes and enable an assessment of differences based on the underlying disease. Additionally, as with many studies, the exact onset of infection for these chronic infections is unknown, and in some patients, the infection appears to precede the first detection by several years.^39^ Therefore, prospective cohorts monitoring enteric viruses in immunocompromised individuals could help to elucidate the early evolutionary dynamics of the virus and to evaluate the effectiveness of expeditious usage of Ig treatment.

## Contributors

RWI-L, conceptualization, data generation and formal analysis, writing—original draft; NV, data generation, writing—review & editing; DAH, treatment of patients, analysis of the clinical data and supplied clinical samples, funding acquisition, writing—review & editing; CMES, next-generation sequencing, writing—review & editing; SRP, data generation, writing—review & editing; DN, data analysis, writing—review & editing; JIJM, data generation, writing—review & editing; IG, resources, writing—review & editing; VASHD, analysis of the clinical data and supplied clinical samples, writing—review & editing; PLAF, treatment of patients, analysis of the clinical data and supplied clinical samples, funding acquisition, writing—review & editing; JJAvK, treatment of patients, analysis of the clinical data and supplied clinical samples, funding acquisition, writing—review & editing; MPGK, funding acquisition, supervision, resources, writing—review & editing; MdG, conceptualization, supervision, formal analysis, funding acquisition, writing—original draft. RWI-L and MdG accessed and verified all the data. All authors have read and approved the final version of the manuscript.

## Data sharing statement

Norovirus genome consensus sequences obtained in this study were submitted under GenBank accession numbers OR536441-OR536499. Raw sequencing files (.fastq) are available on the European Nucleotide Archive (ENA) under Bioproject PRJEB65987. Other relevant information of the samples is available in Supplementary Table S4. All clonal sequences generated in this study are available upon request.

## Declaration of interests

D.A. Hesselink has received grant support, lecture and consulting fees from Astellas Pharma and Chiesi Pharma (paid to his institution). D.A. Hesselink does not have employment or stock ownership at any of these companies, and neither does he have patents nor patent applications.

V.A.S.H. Dalm has received lecture and consulting fees from Pharming, CSL Behring, Takeda, GSK. He has received grant support from Takeda, CSL Behring, AstraZeneca, Moderna and Pharming NV (paid to institution). V.A.S.H. Dalm does not have employment or stock ownership at any of these or other companies, neither does he have patents or patent applications.

P.L.A. Fraaij has received grant support from the Erasmus University Rotterdam, TU Delft, ZonMW (Netherlands) and the European Union's Horizon 2020 program (paid to institution). These institutions did not have any role in the development of this project.

I. Goodfellow has received grant support from the Wellcome trust. This institution did not have any role in the development of this project.

All other authors report no conflict of interest.

## Appendix A Supplementary data

Supplementary Material

Unedited blots
