# Supplementary material for: Patterns of the within-host evolution of human norovirus in immunocompromised individuals and implications for treatment
Source: eBioMedicine. 2024 Oct 12;109:105391. doi: 10.1016/j.ebiom.2024.105391 (PMC11663770; doi:10.1016/j.ebiom.2024.105391)
Supplement: Supplementary Material [file mmc2.pdf]

Supplementary Appendix

Contents

Supplementary Methods 2
RNA extraction and Illumina sequencing 2
Genome assembly and single nucleotide variant (SNV) analysis 2
Haplotype reconstruction 3
Analysis of selection pressures for the GII.4 VP1 codon sites 4
Selection of patients with samples early in the infection 4
Supplementary Figures 5
Supplementary Fig. S1. 5
Supplementary Fig. S2 6
Supplementary Fig. S3 7
Supplementary Fig. S4 8
Supplementary Fig. S5 9
Supplementary Fig. S6 10
Supplementary Fig. S7 11
Supplementary Fig. S8 12
Supplementary Fig. S9 13
Supplementary Fig. S10 14
Supplementary Fig. S11 15
Supplementary Fig. S12 16
Supplementary Fig. S13 17
Supplementary Tables 18
Supplementary Table S1 18
Supplementary Table S2 19
Supplementary Table S3 20
Supplementary Table S5 21
Supplementary Table S6 22
Supplementary Table S7. 23
References 24

Supplementary Methods

RNA extraction and Illumina sequencing

Fecal samples were diluted 1:10 (w/v) in PBS and vortexed. Solid particles were spun down, and the supernatant was treated with Omnicleave endonuclease (Lucigen). RNA was isolated using the High Pure RNA isolation kit (Roche). First-strand synthesis of cDNA was performed using random hexamers and the Superscript IV reverse transcriptase (ThermoFisher Scientific). Second-strand cDNA synthesis was performed using the Klenow fragment. Paired-end libraries were generated using the KAPA HyperPlus kit (Roche), followed by an enrichment step using an *in-house* set of probes, named Gastrocap (designed and synthesized by Roche). GastroCap targets vertebrate gastrointestinal-disease-causing viruses belonging to the *Astroviridae*, *Reoviridae*, *Picornaviridae*, *Adenoviridae*, *Caliciviridae*, *Hepeviridae* and *Parvoviridae* families. After capture of viral reads a PCR amplification step of 14 cycles was performed to increase the concentration of reads. Libraries were sequenced with the 2x250 bp protocol in a Miseq sequencer (Illumina), according to manufacturer instructions.

Genome assembly and single nucleotide variant (SNV) analysis

After Illumina sequencing, FASTQ files containing the raw reads were processed with fastp[1] to remove low-quality or short reads (<50 nts) and trim the low-quality ends. A *de novo* assembly was performed using MetaSpades[2] for samples of day 0. The retrieved near-complete genomes were used as references for the subsequent analysis of patient P18, P19, and P20 samples by a customized workflow in Galaxy EU[3]. Briefly, processed reads were mapped against the genome obtained for day 0 with BWA-MEM[4], generating a BAM file. To improve the alignment of the mapped reads, they were re-aligned in the BAM file using the leftalign utility from FreeBayes package[5]. Both consensus sequences and the SNV analysis were generated from the BAM files using iVar[6]. Final consensus sequences (frequency ≥50%) were constructed using mapped reads at ≥5X coverage and a Phred score ≥30. Consensus genomes were genotyped using the Noronet typing tool[7]. The parameters for the detection of SNVs were set as follows: a minimum coverage of 100X, Phred score ≥30 and a minimum frequency threshold of 1% or 10%. SNVs with a frequency ≥10% were used for the global analyses of the samples. This conservative cutoff was chosen as it gives almost no false positive SNVs[8,9]. For the detection of SNVs with a threshold of 1%, the presence of at least 3 reads containing the specific variant was required. The lower threshold was only used for SNVs confirmed to be present through clonal sequencing and identified as lineage defining mutations. NGS files from the other 17 immunocompromised patients (P1-P17)[8,10] were reanalyzed with the same workflow for SNVs detection. Samples from patients P1-P16 were generated using the SureSelect capture approach for norovirus[8,11], whereas samples of patient P17 were sequenced using the VirCapSeq-VERT capture approach[12]. Sequencing data of a sample was considered of high quality if the genome completeness (the proportion of the genome that has been covered by reads) was ≥95% at 100X

coverage, medium quality if the genome completeness was ≥95% at a 5X coverage and low quality if these parameters were unattained.

For further global analyses of the SNV data, only samples with high quality sequencing data were included. Samples with potential re- or co-infections were dismissed if: i) they did not form a monophyletic group, as seen for samples of patients P2, P3, P4 and P20; ii) any *de novo* assembled contig (>500bp) of a sample was genetically more closely related to other norovirus sequences in GenBank database than to the consensus from the same host, as seen for patients P3, P4 and P10 (determined by BLAST, data not shown); iii) Samples collected after Ig treatment, thus, samples of P18 from day 1138 onwards and samples of P19 from day 1075 onwards were excluded.

The number of cumulative unique emerging SNVs was calculated as the total number of different SNVs present throughout the infection or up to a specific time point. SNVs that were detected in multiple samples of the same host counted as 1. SNVs detected at day 0 were considered as pre-existing SNVs and were subtracted to calculate the number of cumulative unique emerging SNVs. The mutation rate of the genome or a gene was calculated for each time point as follows:

$$mutation\ rate = \frac{SNV_t - SNV_0}{\mathrm{L} \times \mathrm{t}}$$

Where: t is the time in days since day 0 (day where the virus was first detected); SNV_t is the number of unique cumulative SNVs present from the first day of infection up to the time point t of the patient; SNV_0 is the number of unique SNVs at day 0; and, L is the number of nucleotides of the analyzed region.

Haplotype reconstruction

The BAM files containing the mapped reads were further filtered to remove reads with a length below 200 bp. Identical duplicated reads and reads with three or more consecutive N base calls were also discarded. Samples of low sequencing coverage samples were not considered for the analysis. Viral haplotypes of the VP1 were reconstructed using the assembly tool QuasiRecomb 1.2.1 (https://github.com/cbg-ethz/QuasiRecomb/releases)[13,14]. The conservative, quality and refine modes of QuasiRecomb were applied. QuasiRecomb implements a hidden Markov model to infer viral quasispecies haplotypes from deep-coverage NGS data using the expectation maximization algorithm for maximum a posteriori parameter estimation[13,14]. Only haplotypes with an estimated frequency ≥ 1% were used for further analysis. AA pairwise distances among clones and haplotypes derived from each patient were calculated with Mega 11[15], using the JTT matrix-based model[16]. The rate of variation among sites was modeled with a gamma distribution (shape parameter = 1). Multidemensional scaling (MDS) of the AA pairwise distances of the quasispecies of each patient was performed and plotted using the python libraries sklearn and matplotlib.

Analysis of selection pressures on GII.4 VP1 codon sites

To determine which codon positions are under diversifying or purifying selection, the ratio of non-synonymous (d_N) to synonymous (d_S) mutations per codon site was evaluated using the fixed effects likelihood (FEL) and the mixed effects model of evolution (MEME) methods, implemented in the Datamonkey web interface (https://www.datamonkey.org/)[17]. To determine the site-specific pressures of the VP1-encoding sequences, we analyzed the reconstructed haplotypes (≥1% frequency) of the VP1 gene for the viral population within each patient. Only codon positions detected with both methods (default settings) and in at least 2 patients were reported.

Selection of patients with samples early in the infection

To identify patients where the onset of infection was close to the first collected sample, we used two criteria: (i) The estimated date of the onset of infection of the patient should be very close to the date of first sample collection (day 0). Specifically, the 95% highest posterior density (HDP) of the date of the most recent common ancestor (TMRCA) of each patient should overlap with the date of the first collected sample, without signs of re- or co-infections. These parameters were derived from the BEAST analysis; and (ii) the initial number of SNVs (≥10% frequency) on day 0 should be fewer than 10 across the whole genome, as a recent infection would likely not have had sufficient time for the intra-host viral population to diversify significantly. Only the viral populations from patients P1, P6, P11, P13 and P14 met these criteria.

Supplementary Figures

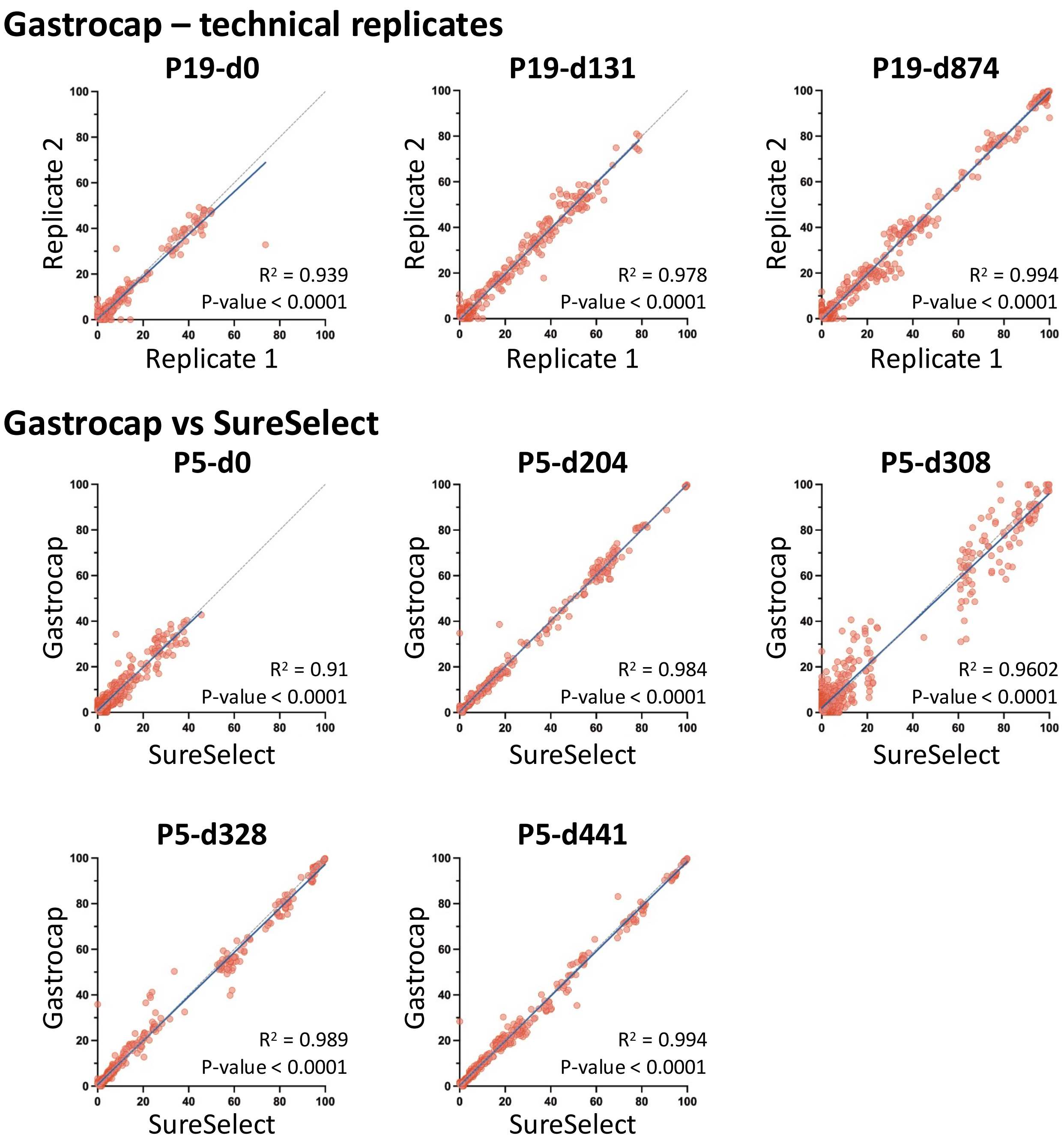

Supplementary Fig. S1. Detection of SNVs by Gastrocap and SureSelect. The top panels show the comparison of SNV frequency detection between two technical sequencing replicates (two independent sequencing runs) using the Gastrocap capture enrichment step for library preparation of three samples from patient P19. The bottom panels present a comparison of SNV frequency detection between the Gastrocap and SureSelect capture approaches for five samples from patient P5. The SureSelect capture approach was previously performed for samples from patients P1-P16. The identity line (dotted diagonal line) and the simple regression line (blue) are shown in each plot. For all samples from the same patient, SNVs were determined by iVar using the initial (day 0) consensus sequence of the patient: P19-d0 (GenBank ID: OR536466) or P5-d0 (GenBank ID: MF140676). Only positions with coverage ≥100X in both runs were compared.

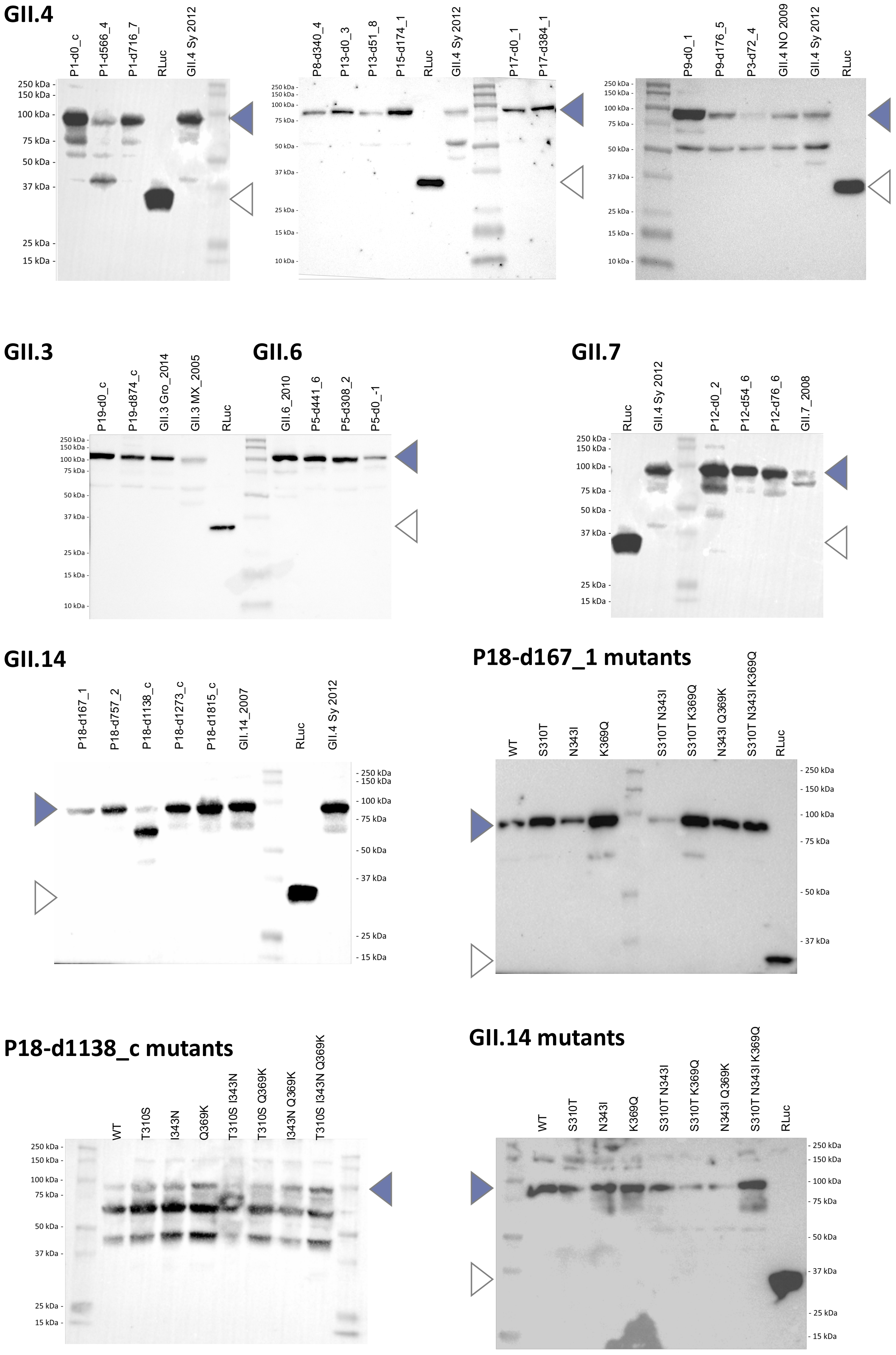

Supplementary Fig. S2. Detection of RLuc-VP1 proteins by Western blot. The RLuc-VP1 proteins in cell lysates were detected by using a polyclonal antibody against the RLuc. Expected sizes: ~98 kDa for RLuc-VP1 proteins (blue triangles) and ~35 kDa for RLuc (white triangles).

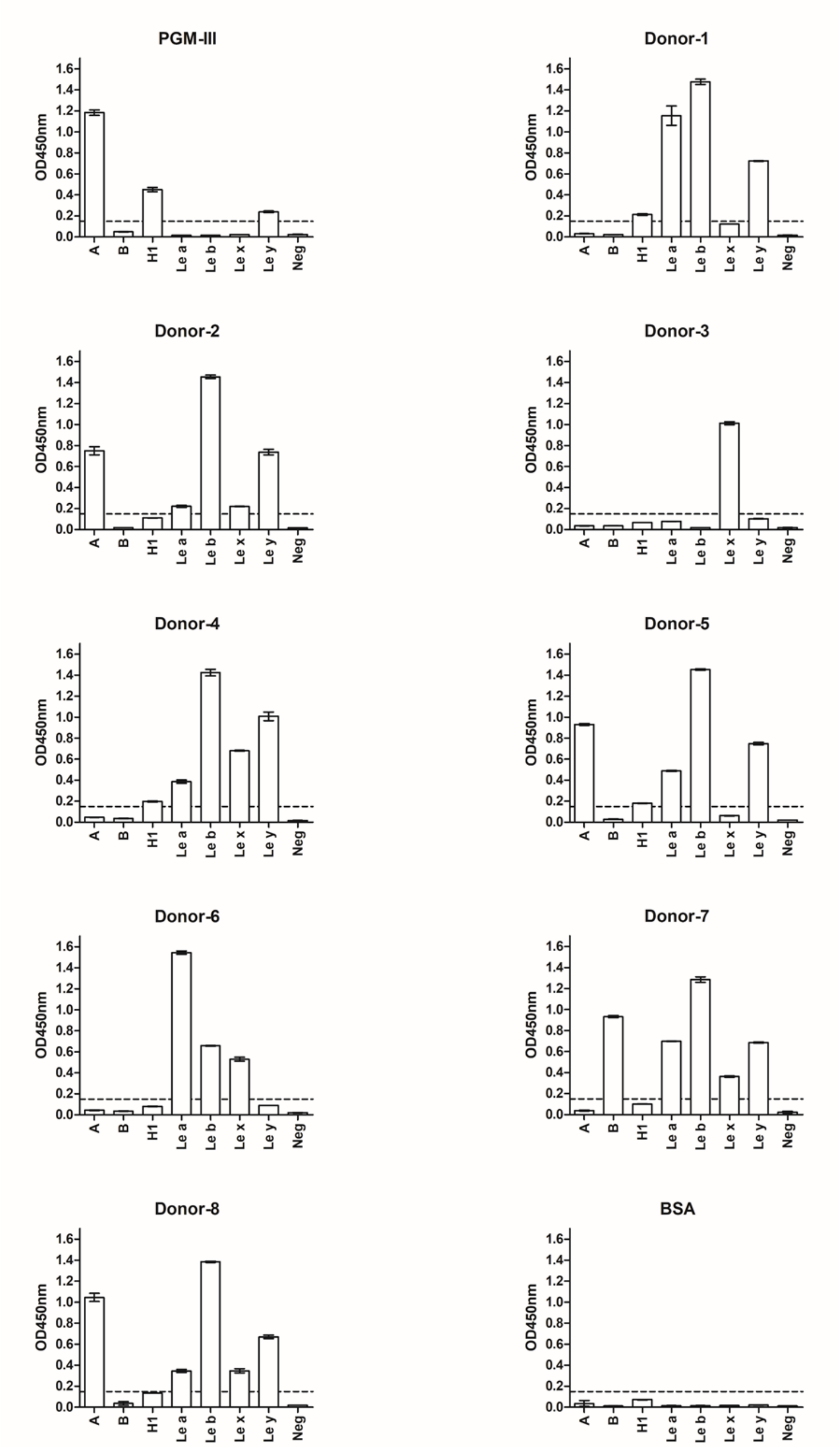

Supplementary Fig. S3. HBGA profile of PGM-III and salivary samples. The presence of specific HBGA was determined by ELISA. PGM-III, saliva samples from eight healthy donors, and BSA were used to coat 96-well plates, and specific glycans were detected using specific antibodies against each glycan. Optical density (OD) signals (450nm) below 0.18 were considered negative.

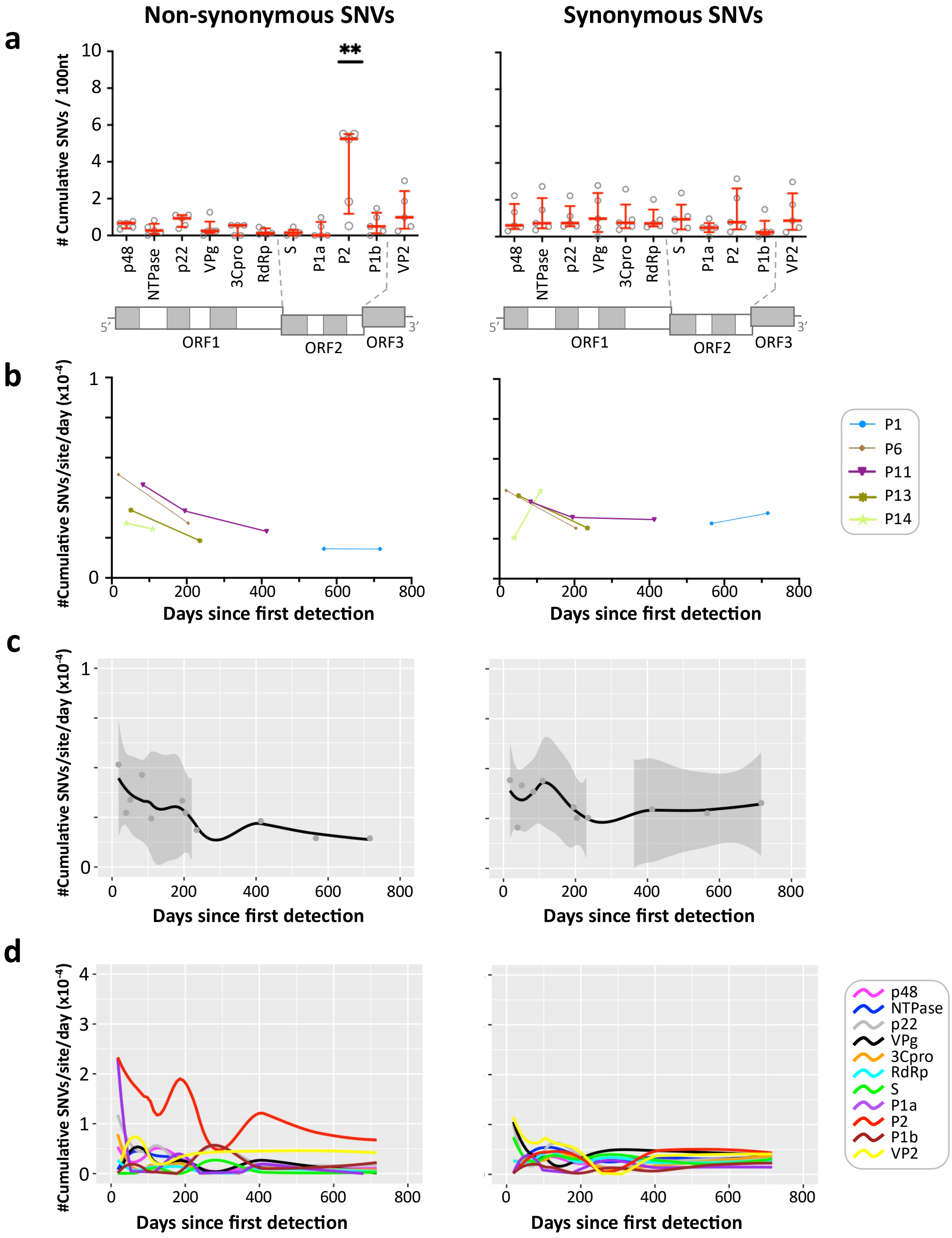

Supplementary Fig. S4. Patterns of evolution of GII.4 in immunocompromised individuals with samples early in the infection (P1, P6, P11, P13, P14) by Single nucleotide variant (SNV) analysis. (a) The number of the cumulative unique emerging SNVs (frequency ≥ 10%) per gene or protein domains by type of mutation (non-synonymous and synonymous). The median and the interquartile range are shown in red. P-values were determined by comparing each gene to the correspondent RdRp. **P<0.01. (b) Rate of mutations over time (cumulative unique emerging SNVs per site per day) in the genome of each individual patient by genotype and type of mutation. (c) Rate of mutations over time in the genome of aggregated patient data by genotype and type of mutation. The curves show LOESS fits, and shaded areas show 95% confidence intervals, as implemented in the geom_smooth function in ggplot2 R package. (d) Rate of mutations over time per gene of aggregated patient data by genotype and type of mutation. Only LOESS curves are shown per gene.

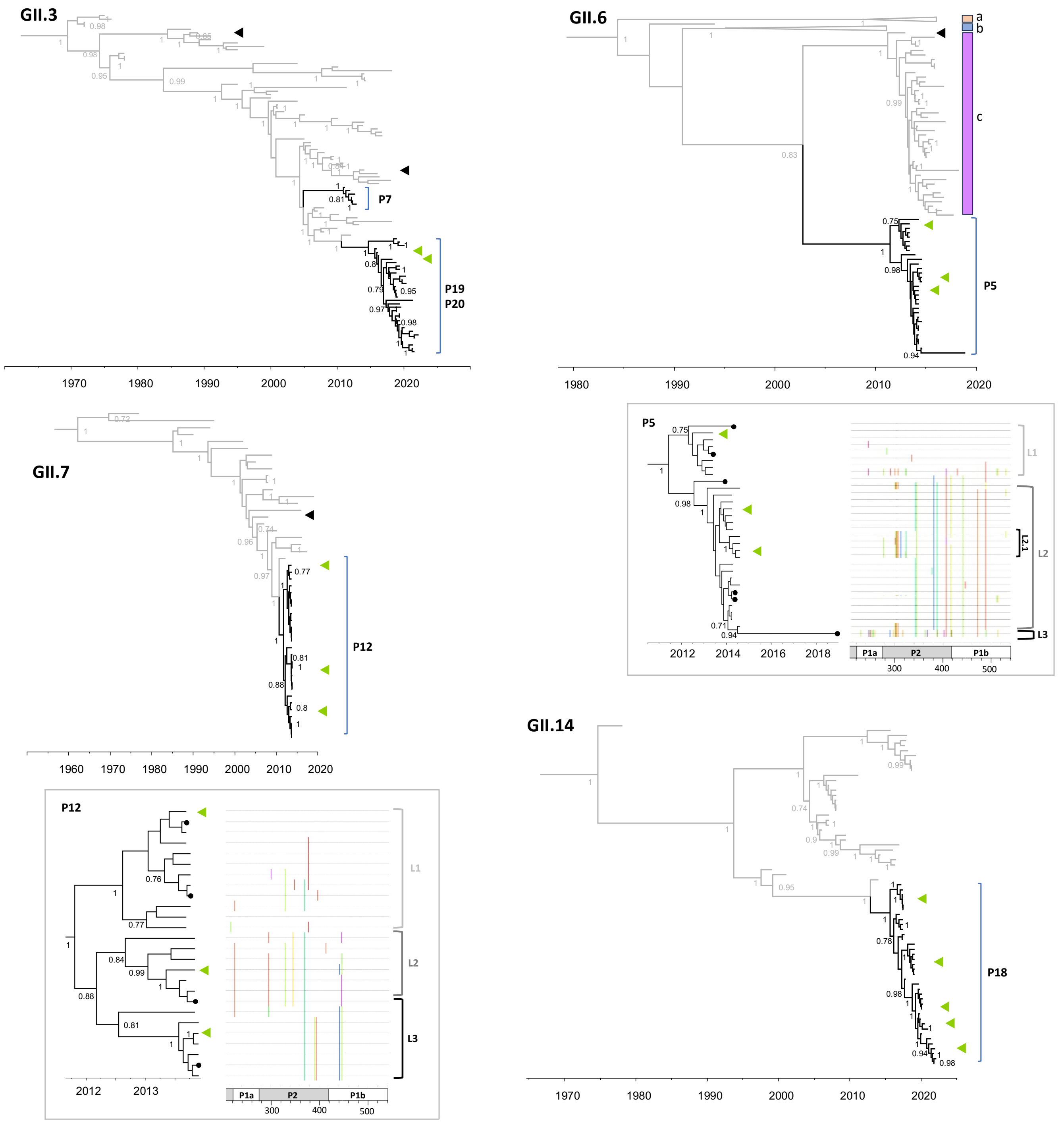

Supplementary Fig. S5. Time-scaled phylogenetic tree for non-GII.4 noroviruses. Trees were constructed from clonal and NGS consensus VP1 nucleotide sequences of norovirus GII.3, GII.6, GII.7 and GII.14 from six chronically infected patients, along with reference sequences. For GII.6 sequences, variants (a-c) are indicated. Patient-derived sequences are indicated in the main tree. Representative patient-derived sequences (green triangles) and reference sequences (black triangles) that were further expressed and characterized are indicated. Consensus sequences are indicated as black circles in the subtrees. Selected posterior values above 0.7 are shown. Specific subtrees show the intra-host norovirus diversity within patients P5 and P12. Matched highlighter plots show AA substitutions in the P-domain relative to the top sequence in the phylogeny.

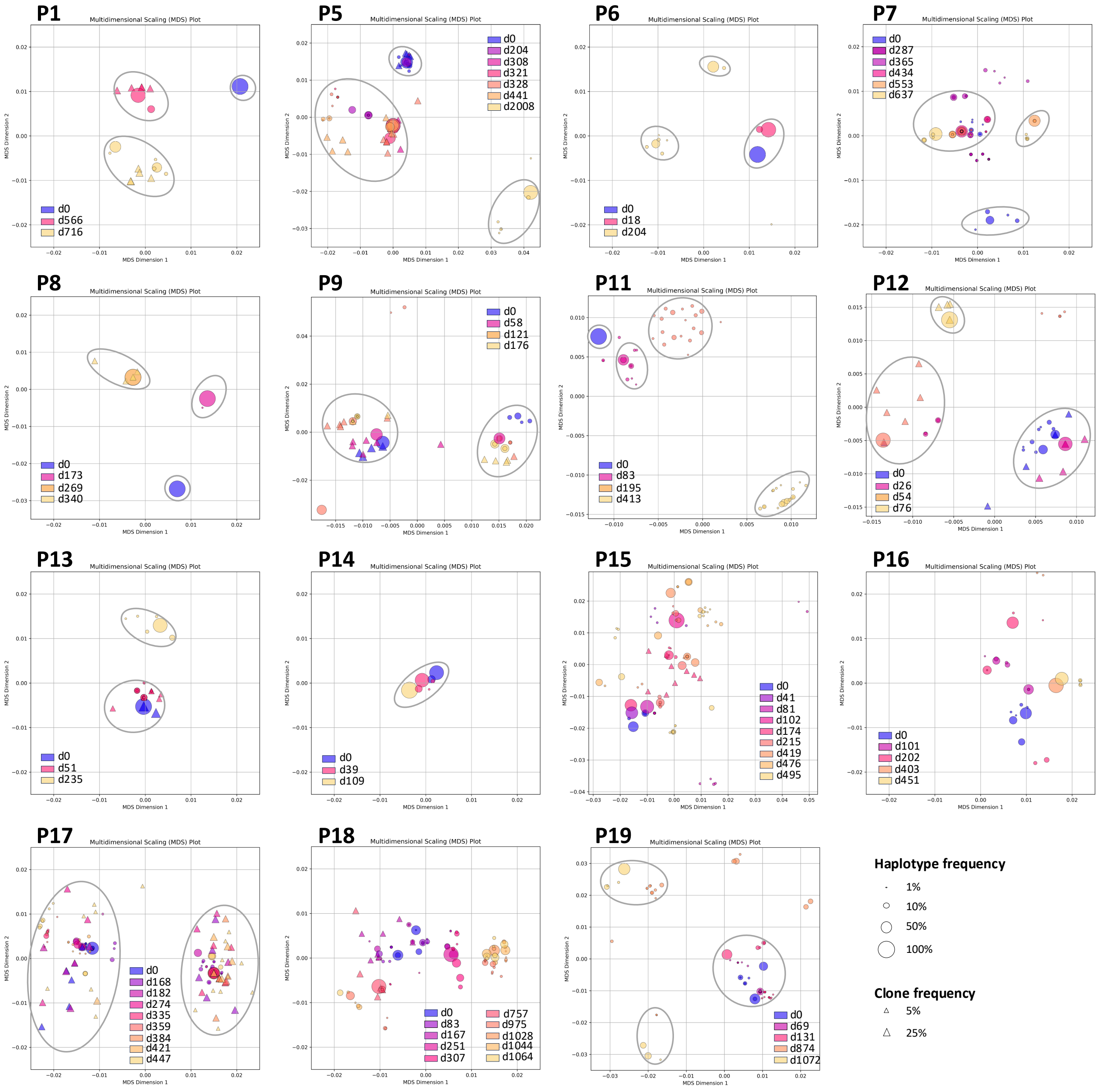

Supplementary Fig. S6. Multidimensional scaling (MDS) of the AA pairwise distances of the quasispecies haplotypes and clones. AA pairwise differences between haplotypes (circles) and clones (triangles) were calculated and used for MDS clustering. The plot shows the first two components of the MDS. Samples are coloured based on time since first detection for each patient. Haplotypes are scaled according to their predicted prevalence as inferred in QuasiRecomb software. Clone sizes are inversely proportional to the number of sequenced clones of the sample (e.g. a clone will be represented as a size corresponding to 20% if the sample from which is derived has a total of 5 sequenced clones). Sequences carrying missense mutations were excluded from analysis.

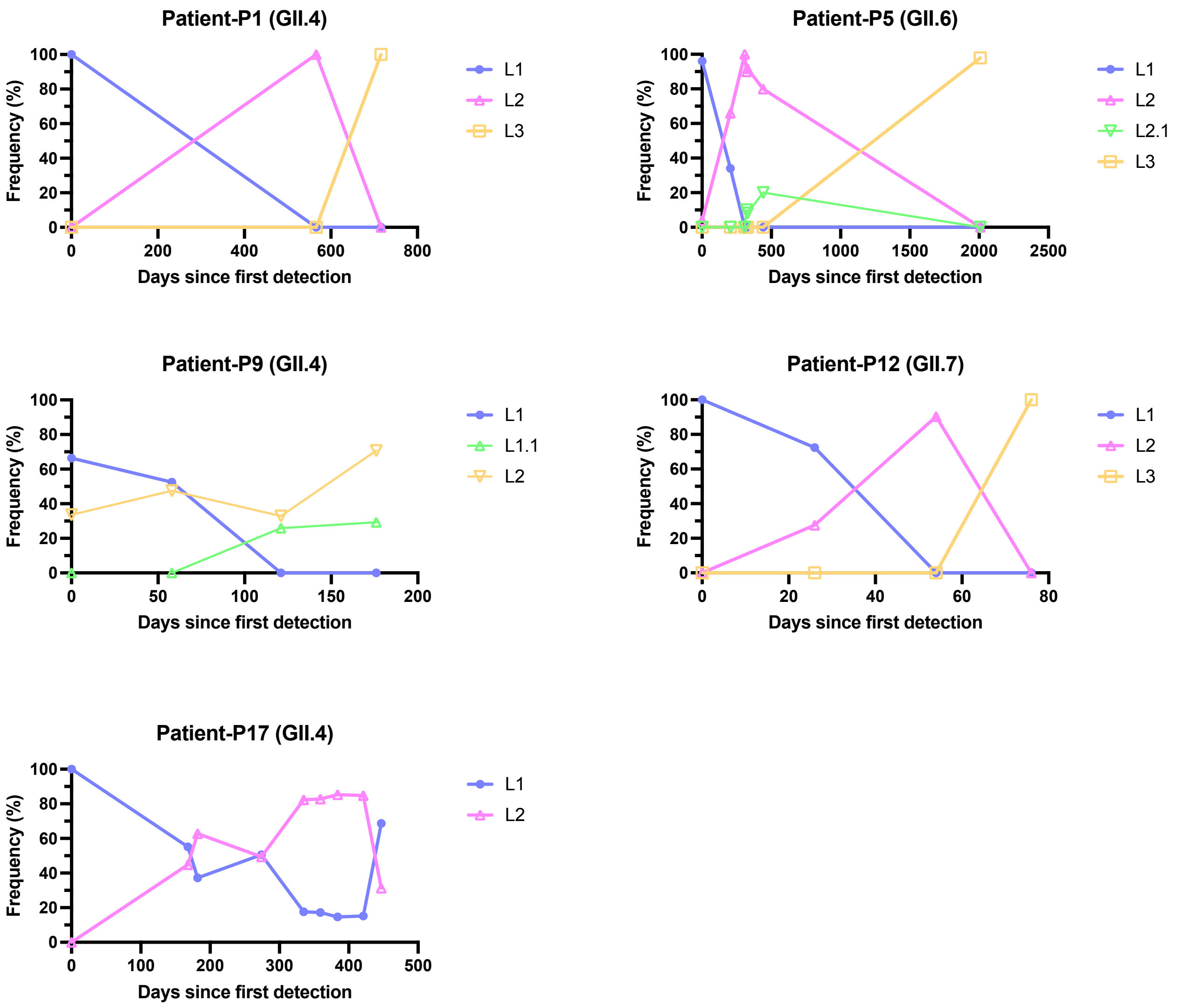

Supplementary Fig. S7. Intra-host lineage dynamics based on the prevalence of the inferred VP1 haplotypes. The frequency of each lineage at any given timepoint was calculated by adding the predicted prevalences of all haplotypes belonging to the lineage in a sample. Haplotypes were inferred with QuasiRecomb. The legend for each plot indicates the lineages detected for the patient.

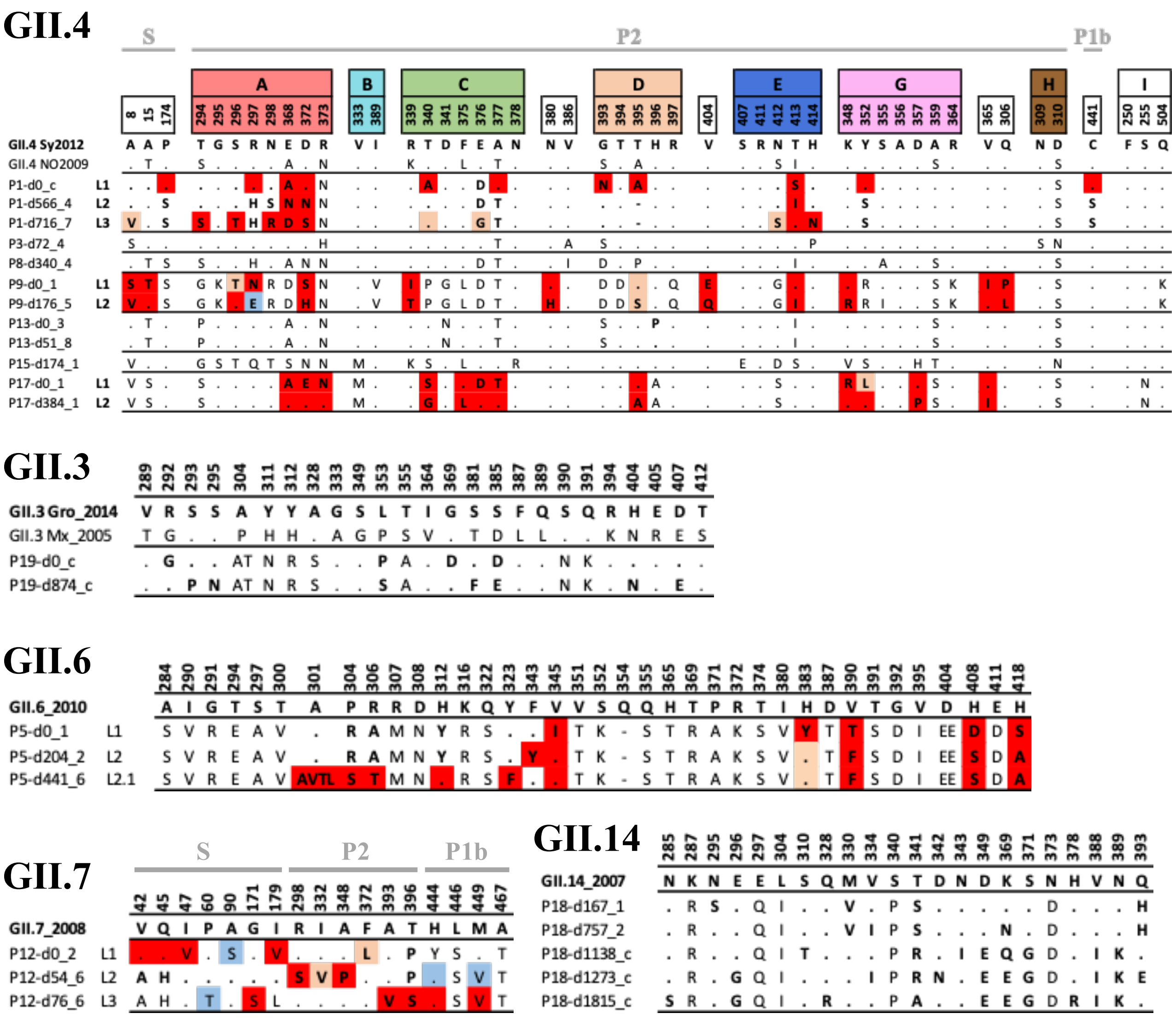

Supplementary Fig. S8. AA differences between expressed VP1 proteins. AA differences in the P2 subdomain of the expressed proteins are shown. When a protein represents an intra-host lineage, the lineage is indicated next to the protein name. Lineage-defining residues between intra-host lineages are highlighted in red. Residues highlighted in orange indicate AA differences that are not lineage-defining mutations, but are present in more than one sequence within the lineage. Residues highlighted in blue indicate AAs unique to that particular clone within the lineage. For GII.4, all residue differences in the P2 domain are indicated if at least one of the expressed VP1 had an AA difference compared to the other GII.4 sequences. AA differences outside of the P2 and Epitope I are shown only if they contained a lineage-defining AA substitution for at least one of the samples. The blockade epitopes are colored based on Fig. 2e. Epitope I is composed of residues located in the P1a (positions 250 and 255) and P1b (position 504). For GII.7s, all AA differences in the VP1 are shown.

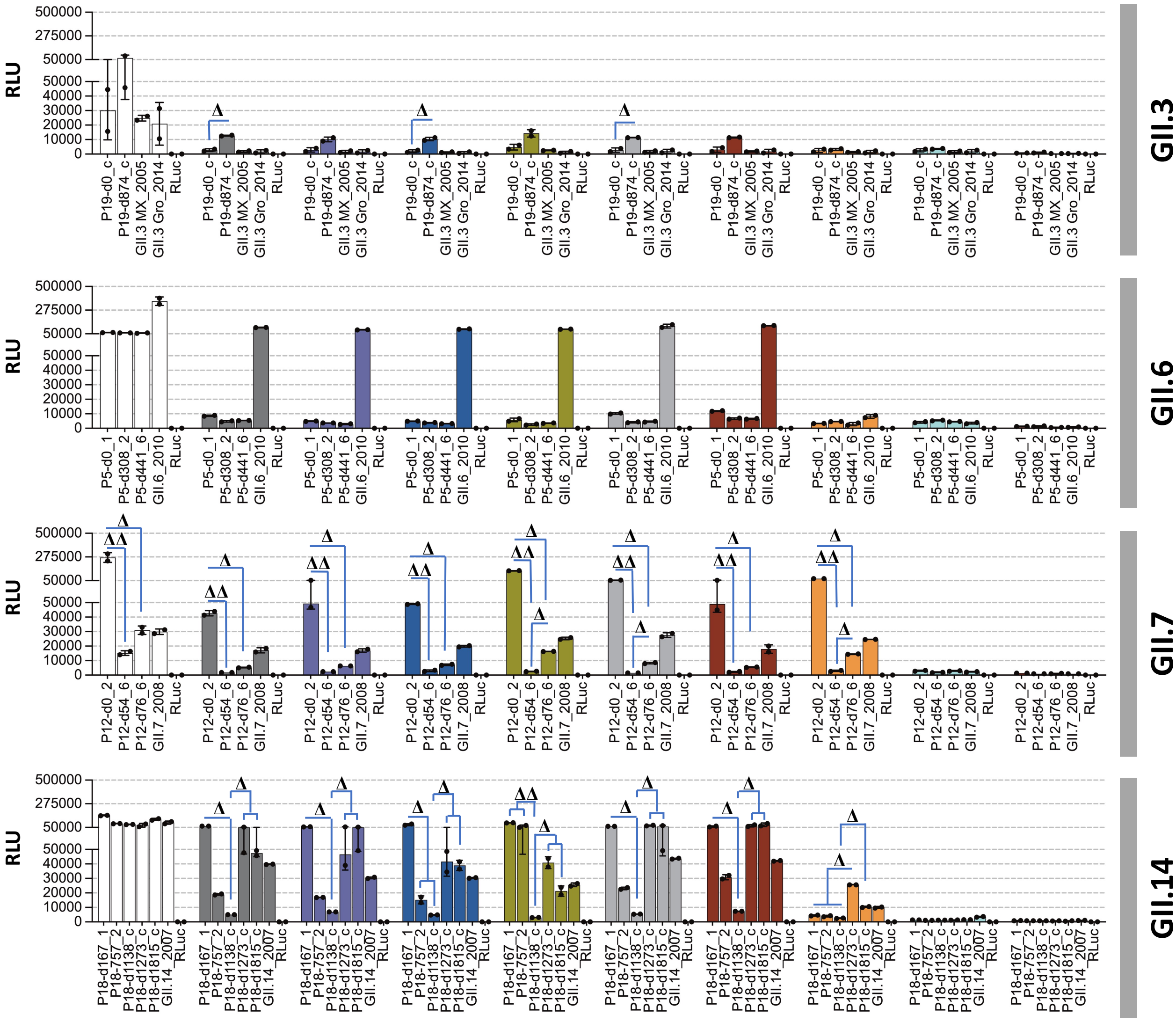

	PGM-III	Donor-2	Donor-5	Donor-8	Donor-7	Donor-1	Donor-4	Donor-6	Donor-3	BSA
A	+++	++	++	+++	-	-	-	-	-	-
B	-	-	-	-	++	-	-	-	-	-
H1	+	-	+	-	-	+	+	-	-	-
Le^a	-	+	+	+	++	+++	+	+++	-	-
Le^b	-	+++	+++	+++	+++	+++	+++	++	-	-
Le^x	-	+	-	+	+	-	++	++	+++	-
Le^y	+	++	++	++	++	++	+++	-	-	-
		A			B	H1			Se$^-$	

Supplementary Fig. S9. Binding of non-GII.4-derived RLuc-VP1 proteins to PGM-III and saliva containing HBGA. The luciferase activity of each RLuc-VP1 protein was adjusted to $5x10^6$ RLU/well before performing the binding assay. Plots are grouped by genotype and HBGA type (bottom table). The average RLU signal ± standard deviation of two independent experiments are shown. The presence of specific HBGAs in the 8 saliva samples (D1-D8) was tested by ELISA (Supplementary Fig. 3). Differences in binding of >4-fold (Δ) and >16-fold (ΔΔ) between intra-host lineages are indicated.

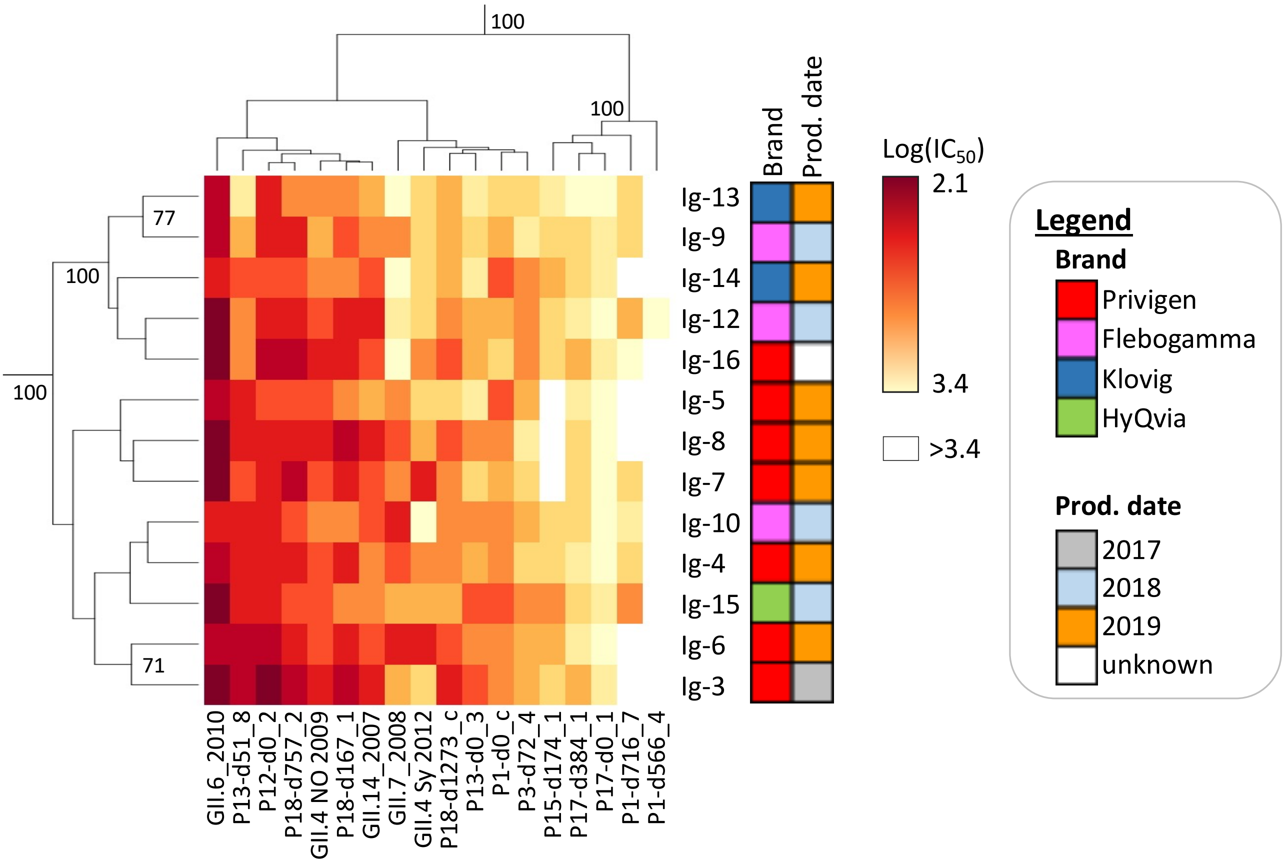

Supplementary Fig. S10. Hierarchical clustering of the tested Ig preparations based on the IC_{50}s of each Ig-antigen combination. Clustering based on the Euclidean distance (Ward.D2 method) and bootstrap values were obtained using the Heatmap hierarchical clustering tool from the HIV Sequence Database (https://www.hiv.lanl.gov/content/sequence/HEATMAP/heatmap.html). Bootstrap values > 70 are shown. Data from Ig-11 was not included given that its blocking activity against many of the RLuc-VP1 proteins could not be tested due to limited amounts. Proteins with IC_{50} values >2500 µg/mL for all Ig preparations were excluded from the analysis.

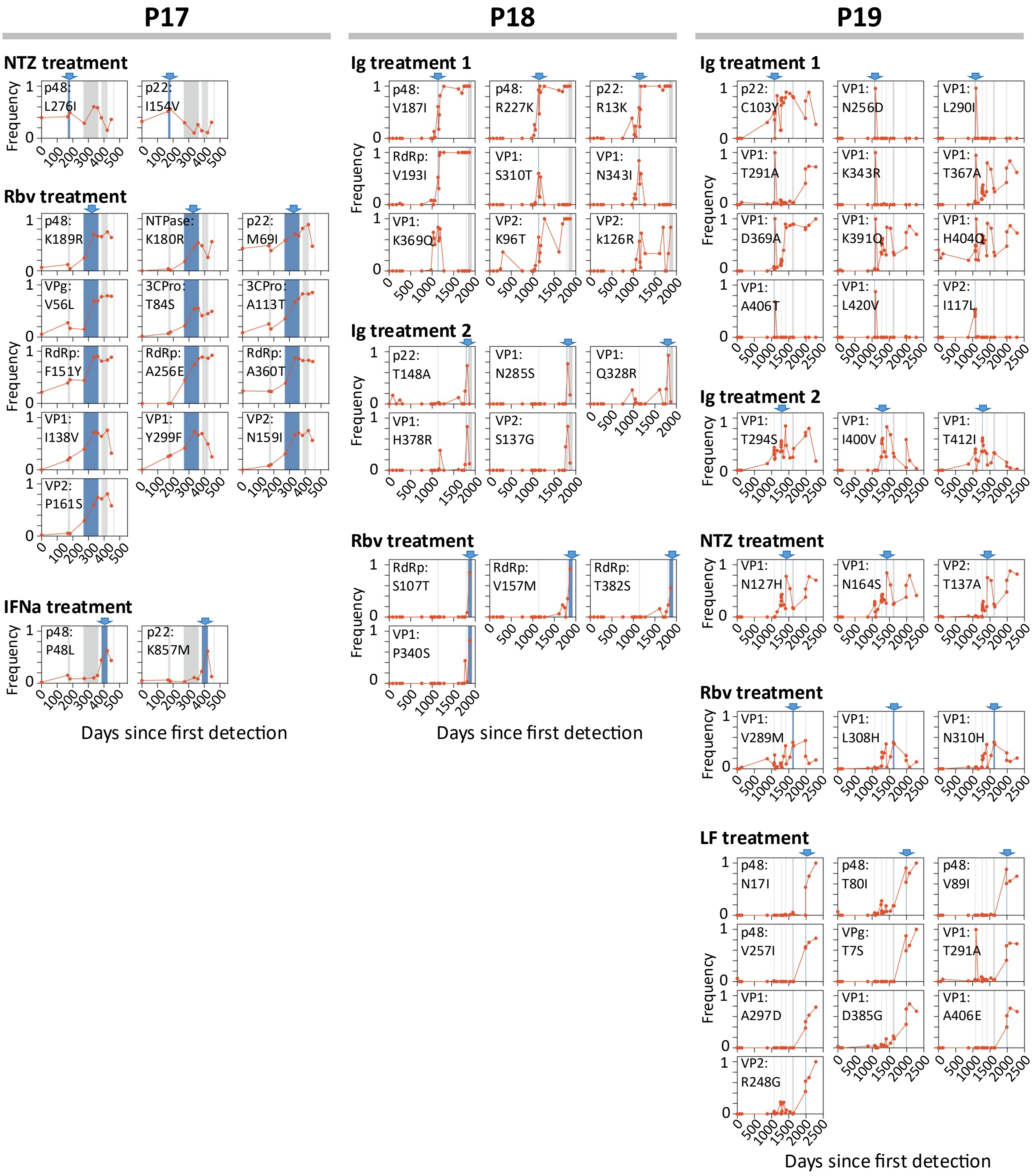

Supplementary Fig. S11. Effects of antiviral treatments on the intra-host prevalence of specific non-synonymous mutations. Mutations that became predominant (≥50% of the reads covering the position) after each of the treatments received by the patients are shown. Blue arrows and blue vertical bars indicate the period during which the specific treatment was given, while gray bars represent the period of the other treatments. Tracing of specific mutations present at ≥1% was considered. The K369Q mutation (patient-P18, Ig treatment 1) is also shown, despite being already dominant before treatment, because it was unique to one of the expressed proteins for this patient.

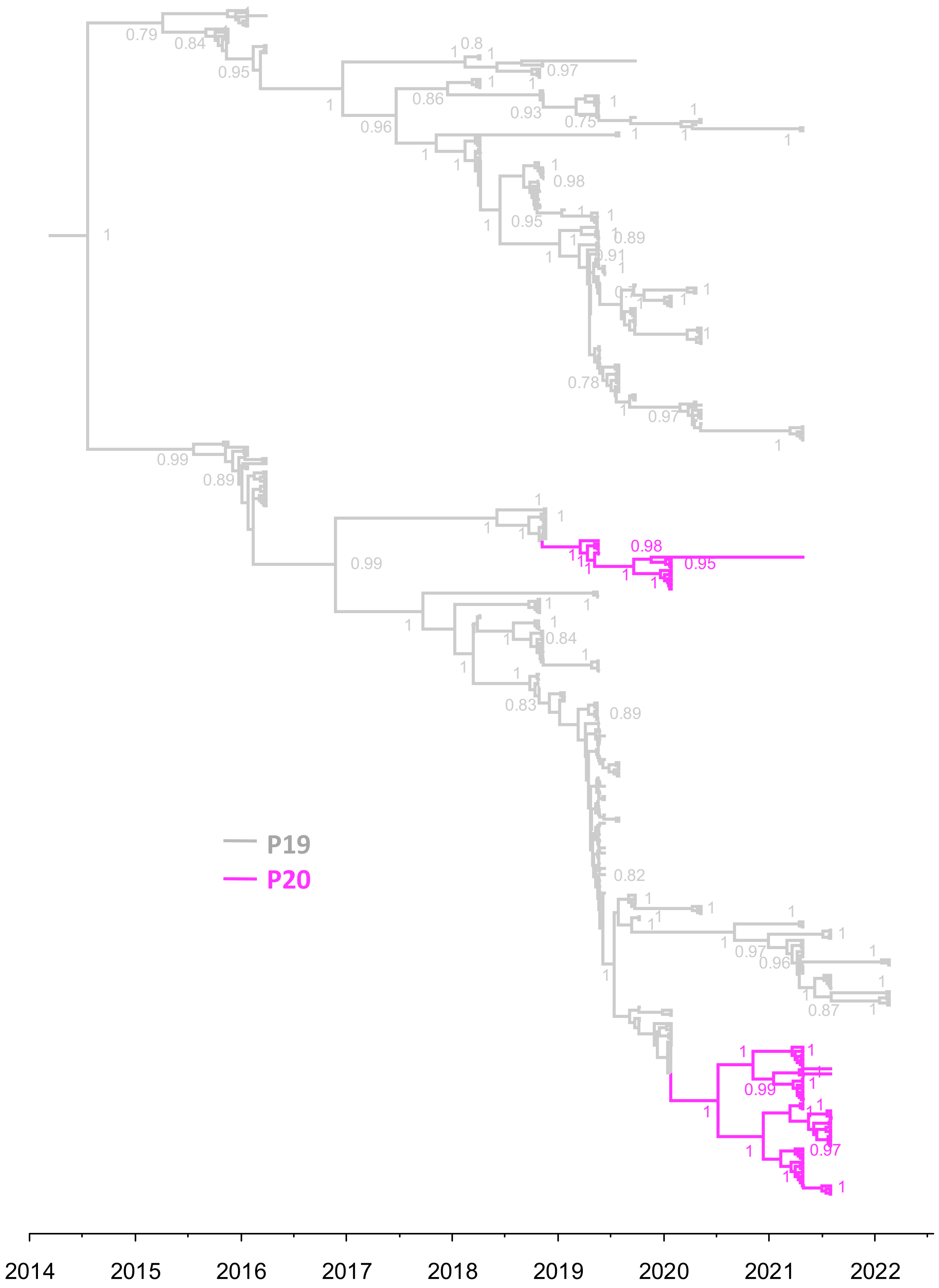

Supplementary Fig. S12. Time-scaled phylogenetic tree of the P19- and P20-derived VP1 haplotype sequences. Haplotype sequences were inferred from NGS reads using QuasiRecomb, and only those with a predicted intra-sample frequency ≥ 1% were used for phylogenetic analysis. Haplotypes for sample P20-d0 could not be predicted due to low coverage of the sample in the NGS data. Posterior values >0.7 are shown.

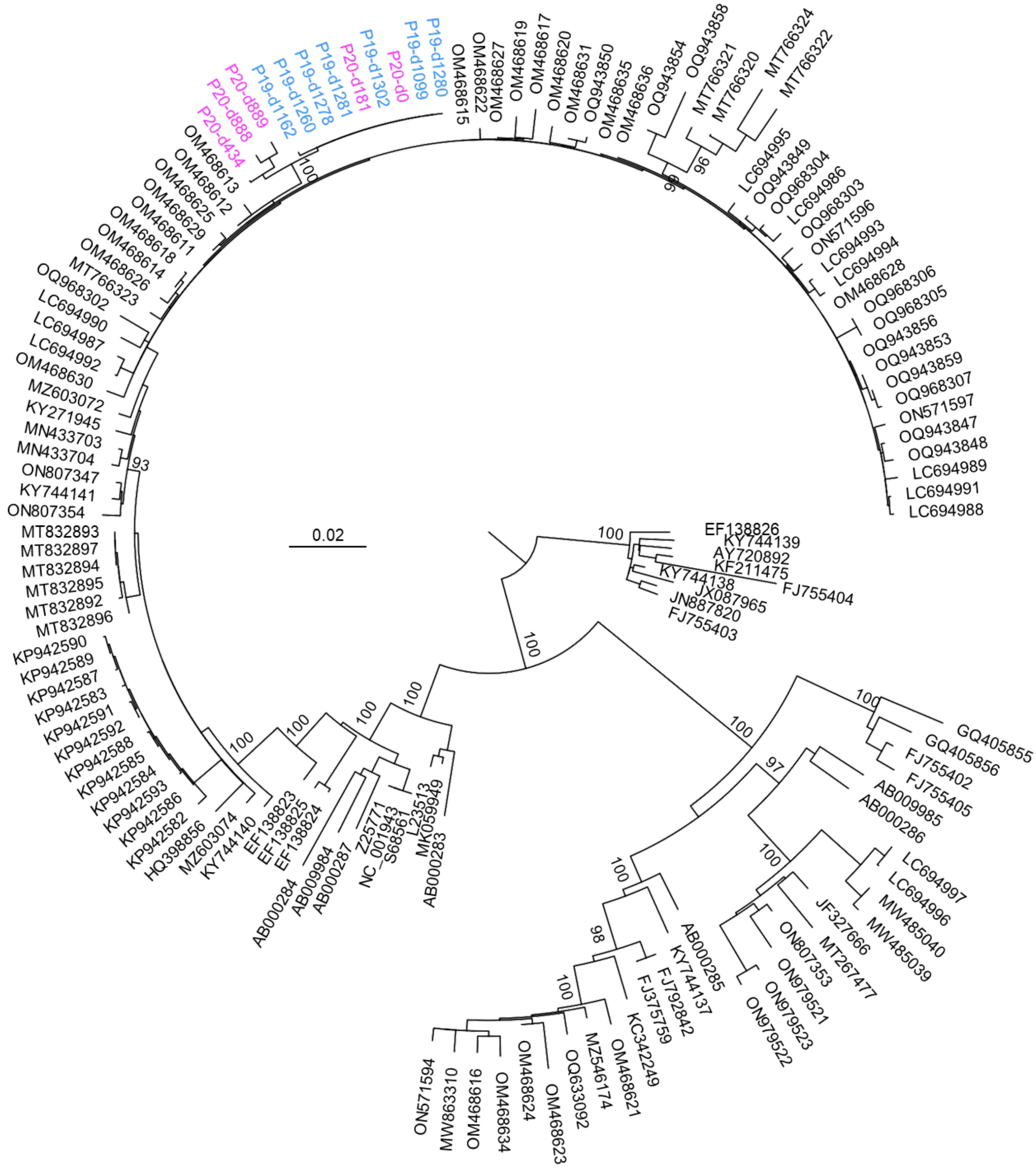

Supplementary Fig. S13. Phylogenetic evidence of person-to-person transmission of HAstV-1 between 2 immunocompromised patients. A Maximum-Likelihood tree was constructed from P19-P20-derived HAstV-1 consensus sequences along with reference sequences (n = 100) from GenBank. The final alignment contains 2350 nucleotide positions corresponding to the HAstV-1 ORF2 gene (capsid). Only samples with complete coverage of this region were included for the alignment. Samples were considered as HAstV-1 positive if at least 0.1% of the reads were mapped to the reference genome and at least 50% of the genome was covered with ≥5X depth.

Supplementary Tables

Supplementary Table S1. Description of the norovirus-infected patients

Patient Identifier	Age (years)	Date of first norovirus detection	Period of collection (days)	Norovirus Genotype	Underlying disease	Reference
**P1**	56	2009-01	716	GII.4 Apeldoorn [P4 Apeldoorn 2007]	Kidney transplantation	van Beek et al. 2017
**P2**	3	2012-03	402	GII.4 New Orleans [P4 New Orleans]	Kidney transplantation	van Beek et al. 2017
**P3**	72	2013-04	122	GII.4 Sydney [P31]	Kidney transplantation	van Beek et al. 2017
**P4**	44	2013-04	355	GII.4 Sydney [P31]	Kidney transplantation	van Beek et al. 2017
**P6**	55	2011-11	204	GII.4 Den Haag 2006b [P4 Den Haag 2006b]	Kidney transplantation	van Beek et al. 2017
**P8**	6	2012-06	340	GII.4 Sydney [P4 New Orleans]	Kidney transplantation	van Beek et al. 2017
**P9**	58	2013-08	176	GII.4 New Orleans [P4 (not assigned)]	Lung transplantation	van Beek et al. 2017
**P10**	57	2013-01	462	GII.4 Sydney [P31]	Lung transplantation	van Beek et al. 2017
**P11**	35	2013-01	413	GII.4 New Orleans [P4 New Orleans]	Allo-HSCT	van Beek et al. 2017
**P13**	57	2010-12	235	GII.4 New Orleans [P4 New Orleans]	ASCT	van Beek et al. 2017
**P14**	48	2008-01	109	GII.4 Den Haag 2006b [P4 Den Haag 2006b]	CLL	van Beek et al. 2017
**P15**	71	2009-06	495	GII.4 Hunter 2004 [P4 (not assigned)]	Good syndrome	van Beek et al. 2017
**P16**	62	2010-06	451	GII.4 not assign [P4 (not assign)]	Vasculitis	van Beek et al. 2017
**P17**	42	2017-03	447	GII.4 New Orleans [P31]	CVID	van Kampen et al. 2022
**P5**	66	2013-05	2008	GII.6 [P7]	Kidney transplantation	van Beek et al. 2017; this study
**P7**	12	2011-02	637	GII.3 [P21]	Kidney transplantation	van Beek et al. 2017
**P12**	36	2013-07	76	GII.7 [P7]	Follicular non-Hodgkin lymphoma	van Beek et al. 2017
**P18**	47	2016-12	1891	GII.14 [P(could not assign)]	CVID / Kidney transplantation	This study
**P19**	3	2015-11	2288	GII.3 [P21]	Agammaglobulinemia	This study
**P20**	0	2018-11	1189	GII.3 [P21]	Agammaglobulinemia	This study

Allo-HSCT = Allogeneic hematopoietic stem cell transplantation
ASCT = Autologous stem cell transplantation
CLL = Chronic lymphocytic leukemia
CVID = Common variable immunodeficiency

Supplementary Table S2. Antibodies used to detect HBGAs in saliva samples

Antibody	Catalog number	Brand	Dilution
**Anti-A (ABO1)**	9113D10	Diagast	1:10
**Anti-B (ABO2)**	9621A8	Diagast	1:10
**Anti-A, B (ABO3)**	9113D10 + 152D12	Diagast	1:10
**Blood group Antigen H (O) Type 1 antibody (17-206)**	14-9810-82	Thermo Fisher	1:200
**Monoclonal Anti-Blood group Lewis A**	SAB4700762-100UG	Sigma-Aldrich	1:400
**Monoclonal Anti-Blood group Lewis B**	SAB4700761-100UG	Sigma-Aldrich	1:400
**mouse Anti-LeY [H18A] IgG1**	AB00493-1.1	Absolute antibody	1:400
**SSEA1 monoclonal antibody MC-480 (Lewis x)**	MA1-022	Thermo Fisher	1:400
**Rabbit Anti-Mouse Immunoglobulins/HRP**	P0260	Dako	1:500

Supplementary Table S3. Ig preparations used in this study

Code	Company	Commercial name	Ig (mg/mL)	Lot nr	Prod. Date	Marketing authorization number
**Ig-3***	CSL Behring GmbH	Privigen	100	P100020808	2017-02	EU/1/08/446/001
**Ig-4**	CSL Behring GmbH	Privigen	100	P100122002	2019-06	EU/1/08/446/003
**Ig-5**	CSL Behring GmbH	Privigen	100	P100093823	2019-04	EU/1/08/446/002
**Ig-6**	CSL Behring GmbH	Privigen	100	P100122104	2019-07	EU/1/08/446/001
**Ig-7**	CSL Behring GmbH	Privigen	100	P100113245	2019-06	EU/1/08/446/002
**Ig-8**	CSL Behring GmbH	Privigen	100	P100122002	2019-06	EU/1/08/446/003
**Ig-9**	Grifols	Flebogamma® DIF	50	A4GFD0051	2018-12	EU/1/07/404/001
**Ig-10**	Grifols	Flebogamma® DIF	50	A4GEC01301	2018-11	EU/1/07/404/001
**Ig-11**	Grifols	Flebogamma® DIF	50	A4GFD0051	2018-12	EU/1/07/404/001
**Ig-12**	Grifols	Flebogamma® DIF	50	A4GDC00601	2018-11	EU/1/07/404/001
**Ig-13**	Baxter AG	Kiovig	100	LE12V091AG	2019-03	EU/1/05/329/005
**Ig-14**	Baxter AG	Kiovig	100	LE12V206AF	2019-08	EU/1/05/329/004
**Ig-15**	Baxalta Innorovirusations GmbH	HyQvia	100	LE16V069AB	2018-07	EU/1/13/840/004
**Ig-16**	CSL Behring GmbH	Privigen	100	Unknown	Unknown	EU/1/08/446/002

*Ig preparation used for the second Ig treatment of patient P19

Supplementary Table S5. Estimated date of infection and initial number of SNVs detected at day 0 for immunocompromised patients with no signs of re- or co-infection in this study.

Patient ID	Date of 1st collection	ORF2 TMRCA(i) [95% HPD interval]	Number of SNV(ii) at day 0
**P1**	2009.0	2008.6 [2008.0 - 2009.0]	1
**P5**	2013.4	2011.3 [2009.5 - 2012.8]	100
**P6**	2011.9	2011.6 [2011.2 - 2011.9]	8
**P7**	2011.1	2010.8 [2010.0 - 2011.1]	110
**P8**	2012.5	2010.9 [2010.6 - 2011.3]	4
**P9**	2013.6	2011.6 [2010.7 - 2012.4]	116
**P11**	2013.1	2012.8 [2012.3 - 2013.1]	0
**P12**	2013.6	2011.6 [2010.8 - 2012.4]	41
**P13**	2010.5	2010.4 [2009.9 - 2010.8]	2
**P14**	2008.1	2007.8 [2007.3 - 2008.1]	5
**P15**	2009.4	2007.9 [2006.8 - 2008.8]	117
**P16**	2010.5	2009.6 [2008.7 - 2010.3]	65
**P17**	2017.2	2014.2 [2013.4 - 2015.2]	333
**P18**	2016.9	2015.5 [2014.5 - 2016.4]	61
**P19**	2015.9	2014.5 [2013.0 - 2015.8]	76

(i)TMRCA: time to the most recent common ancestor; HDP: highest posterior density. These parameters were calculated from BEAST analysis.

(ii)SNV: single nucleotide variants, calculated from the comparison of Illumina read data against the norovirus consensus sequence from the patient at day 0.

Supplementary Table S6. Number of AA changes in ORF2 and ORF3 consensus sequences at the within-host level during chronic infections. AA positions that changed at the consensus level in any timepoint compared to the initial sequence within a patient are counted as 1, regardless of the number of AA changes in that residue position. AA positions are given with respect to a GII.4 Sydney 2012 VP1 reference sequence (JX459908).

GII.4 ORF	AA position	nr consensus changes
**ORF2**	294	3
**ORF2**	297	5
**ORF2**	298	3
**ORF2**	299	3
**ORF2**	340	4
**ORF2**	341	5
**ORF2**	357	3
**ORF2**	365	4
**ORF2**	368	3
**ORF2**	372	9
**ORF2**	373	5
**ORF2**	376	3
**ORF2**	393	6
**ORF2**	395	4
**ORF2**	404	3
**ORF2**	413	4
**ORF3**	5	3
**ORF3**	15	3

Supplementary Table S7. Positive (diversifying) and negative (purifying) selection detected at the intra-host level during chronic infections. Only codon positions where either diversifying or purifying selection was found for two or more patients are given. Predicted haplotypes with a frequency ≥1% from samples of patients with no signs of re- or co-infection were included in the analysis. Codon sites are given with respect to a GII.4 Sydney 2012 VP1 reference sequence (JX459908).

Selection type	VP1 region	Codon site	Nr of detected cases
Diversifying	P2	299	2
Diversifying	P2	331	2
Diversifying	P2	341	2
Diversifying	P2	352	2
Diversifying	P2	359	2
Diversifying	P2	372	3
Diversifying	P2	413	2
Diversifying	P1b	534	2
Purifying	S	54	2
Purifying	S	64	2
Purifying	S	86	2
Purifying	S	118	2
Purifying	S	128	2
Purifying	S	144	2
Purifying	S	163	2
Purifying	S	164	2
Purifying	S	168	3
Purifying	S	189	3
Purifying	S	205	2
Purifying	S	212	2
Purifying	P1a	227	2
Purifying	P1a	239	3
Purifying	P1a	260	2
Purifying	P2	310	2
Purifying	P2	320	2
Purifying	P2	325	3
Purifying	P2	326	2
Purifying	P2	343	2
Purifying	P2	356	2
Purifying	P1b	426	3
Purifying	P1b	475	3
Purifying	P1b	476	2
Purifying	P1b	481	2
Purifying	P1b	488	2
Purifying	P1b	489	3
Purifying	P1b	521	2
Purifying	P1b	522	2

References

1 Chen S, Zhou Y, Chen Y, Gu J. fastp: an ultra-fast all-in-one FASTQ preprocessor. *Bioinformatics* 2018; **34**: i884–90.

2 Nurk S, Meleshko D, Korobeynikov A, Pevzner PA. metaSPAdes: a new versatile metagenomic assembler. *Genome Res* 2017; **27**: 824–34.

3 Afgan E, Baker D, Batut B, *et al.* The Galaxy platform for accessible, reproducible and collaborative biomedical analyses: 2018 update. *Nucleic Acids Res* 2018; **46**: W537–44.

4 Li H. Aligning sequence reads, clone sequences and assembly contigs with BWA-MEM. *arXiv:13033997 [q-bio]* 2013; published online May 26. http://arxiv.org/abs/1303.3997 (accessed July 16, 2020).

5 Garrison E, Marth G. Haplotype-based variant detection from short-read sequencing. *arXiv:12073907 [q-bio]* 2012; published online July 20. http://arxiv.org/abs/1207.3907 (accessed July 16, 2020).

6 Grubaugh ND, Gangavarapu K, Quick J, *et al.* An amplicon-based sequencing framework for accurately measuring intrahost virus diversity using PrimalSeq and iVar. *Genome Biol* 2019; **20**: 8.

7 Kroneman A, Vennema H, Deforche K, *et al.* An automated genotyping tool for enteroviruses and noroviruses. *Journal of Clinical Virology* 2011; **51**: 121–5.

8 van Beek J, de Graaf M, Smits S, *et al.* Whole-Genome Next-Generation Sequencing to Study Within-Host Evolution of Norovirus (NoV) Among Immunocompromised Patients With Chronic NoV Infection. *J Infect Dis* 2017; **216**: 1513–24.

9 Tohma K, Saito M, Pajuelo MJ, *et al.* Viral intra-host evolution in immunocompetent children contributes to human norovirus diversification at the global scale. *Emerg Microbes Infect* 2021; **10**: 1717–30.

10 van Kampen JJA, Dalm VASH, Fraaij PLA, *et al.* Clinical and In Vitro Evidence Favoring Immunoglobulin Treatment of a Chronic Norovirus Infection in a Patient With Common Variable Immunodeficiency. *J Infect Dis* 2022; **226**: 1781–9.

11 Brown JR, Roy S, Ruis C, *et al.* Norovirus Whole-Genome Sequencing by SureSelect Target Enrichment: a Robust and Sensitive Method. *J Clin Microbiol* 2016; **54**: 2530–7.

12 Briese T, Kapoor A, Mishra N, *et al.* Virome Capture Sequencing Enables Sensitive Viral Diagnosis and Comprehensive Virome Analysis. *mBio* 2015; **6**: 10.1128/mbio.01491-15.

13 Afridi SQ, Usman Z, Donakonda S, *et al.* Prolonged norovirus infections correlate to quasispecies evolution resulting in structural changes of surface-exposed epitopes. *iScience* 2021; **24**: 102802.

14 Töpfer A, Zagordi O, Prabhakaran S, Roth V, Halperin E, Beerenwinkel N. Probabilistic inference of viral quasispecies subject to recombination. *J Comput Biol* 2013; **20**: 113–23.

15 Tamura K, Stecher G, Kumar S. MEGA11: Molecular Evolutionary Genetics Analysis Version 11. *Mol Biol Evol* 2021; **38**: 3022–7.

16 Jones DT, Taylor WR, Thornton JM. The rapid generation of mutation data matrices from protein sequences. *Comput Appl Biosci* 1992; **8**: 275–82.

17 Weaver S, Shank SD, Spielman SJ, Li M, Muse SV, Kosakovsky Pond SL. Datamonkey 2.0: A Modern Web Application for Characterizing Selective and Other Evolutionary Processes. *Molecular Biology and Evolution* 2018; **35**: 773–7.
