# Supplementary material for: Patterns of the within-host evolution of human norovirus in immunocompromised individuals and implications for treatment
Source: eBioMedicine. 2024 Oct 12;109:105391. doi: 10.1016/j.ebiom.2024.105391 (PMC11663770; doi:10.1016/j.ebiom.2024.105391)
Supplement: Unedited blots [file mmc3.pdf]

Full unedited blot for Supplemental Figure 6

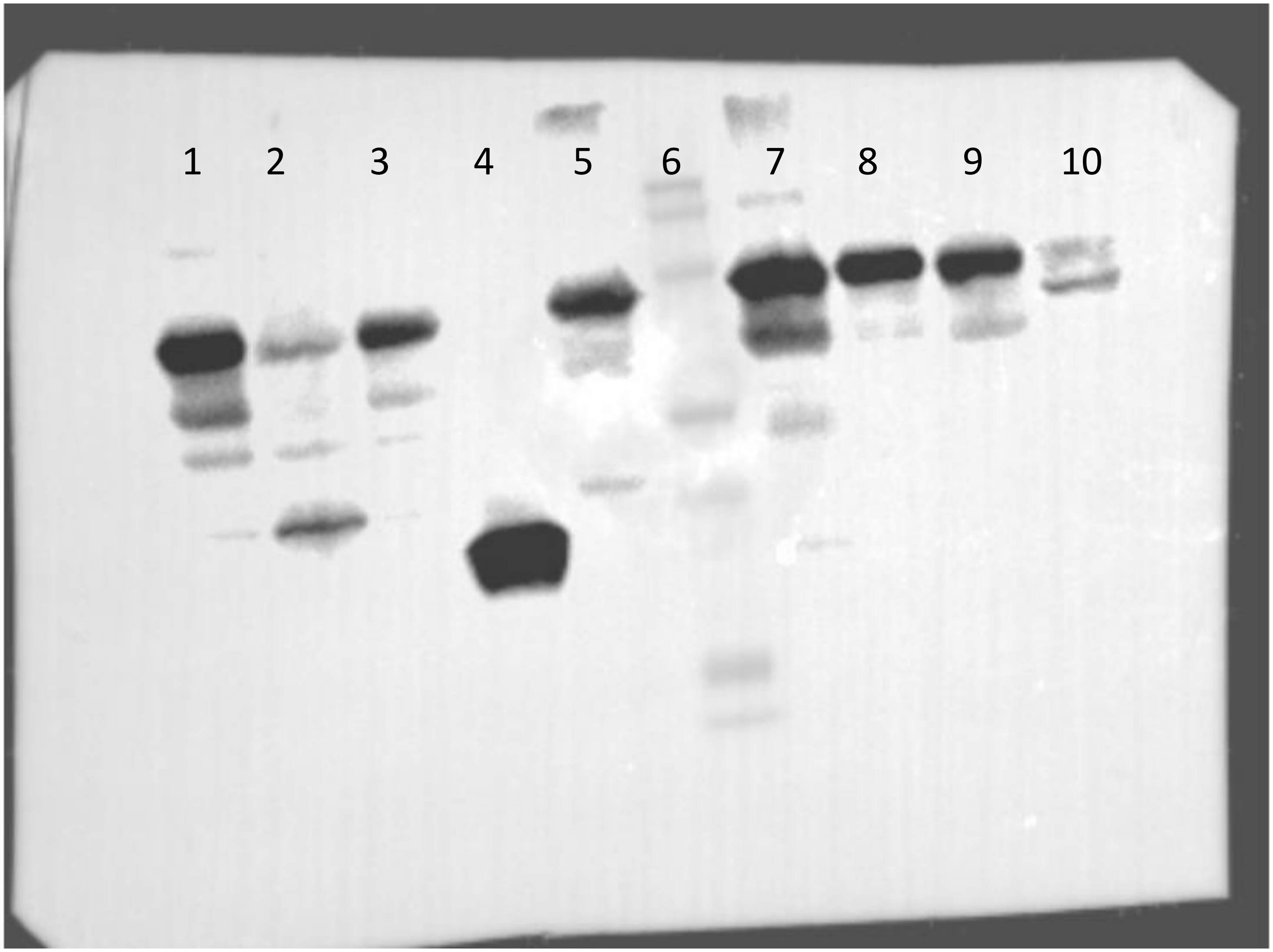

Lane	Protein	Location in Supplemental Figure 6
1	P1-d0_c	Lane 1 in the left panel for GII.4 blots.
2	P1-d566_4	Lane 2 in the left panel for GII.4 blots.
3	P1-d716	Lane 3 in the left panel for GII.4 blots.
4	Rluc	Lane 4 in the left panel for GII.4 blots. Lane 1 in the GII.7 panel.
5	GII.4 Sy 2012	Lane 5 in the left panel for GII.4 blots. Lane 2 in the GII.7 panel.
6	Ladder	Lane 6 in the left panel for GII.4 blots. Lane 3 in the GII.7 panel.
7	P12-d0_2	Lane 4 in the GII.7 panel.
8	P12-d54_6	Lane 5 in the GII.7 panel.
9	P12-d76_4	Lane 6 in the GII.7 panel.
10	GII.7_2008	Lane 7 in the GII.7 panel.

Full unedited blot for Supplemental Figure 6

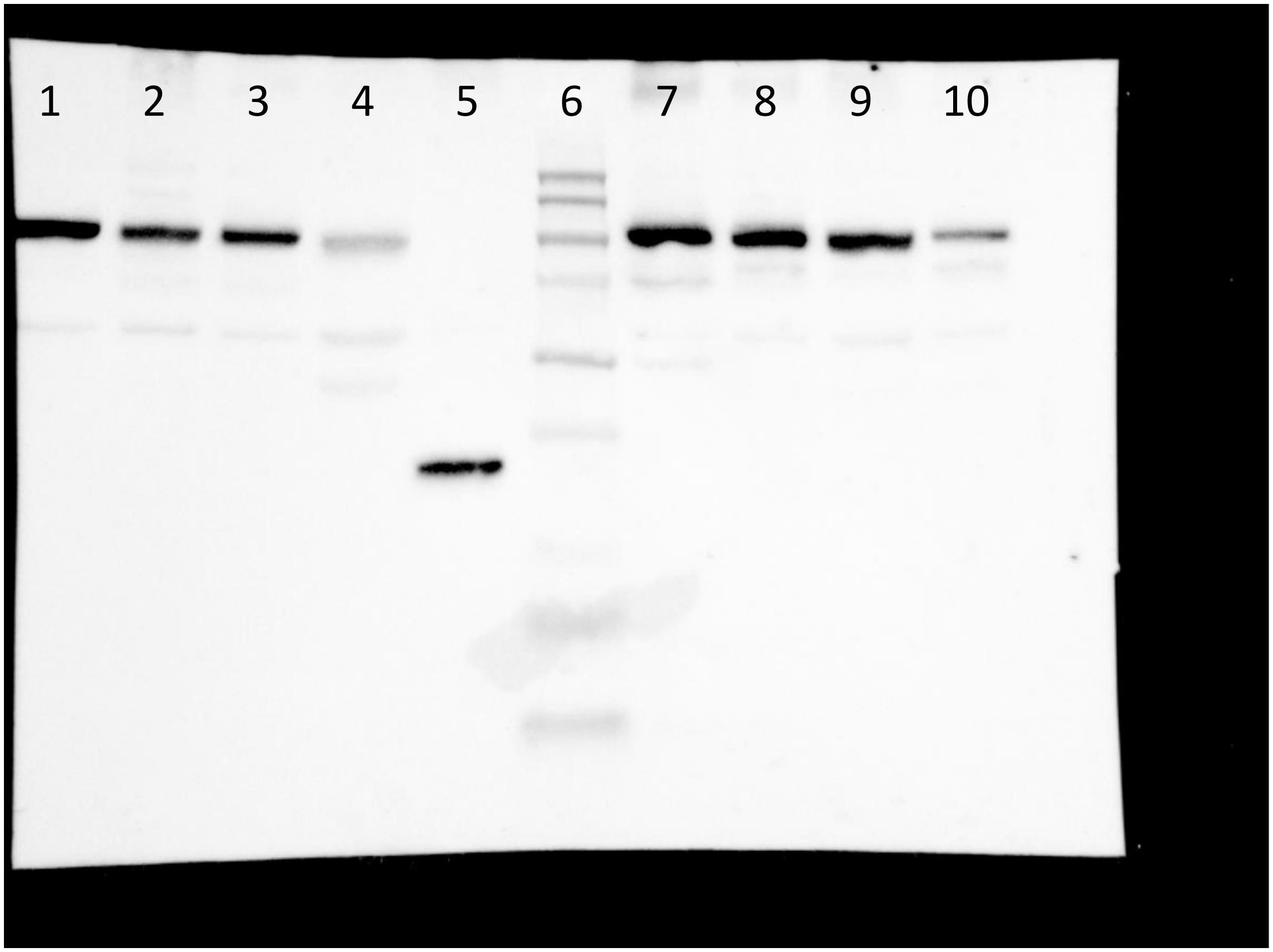

Lane	Protein	Location in Supplemental Figure 6
1	P19-d0_c	Lane 1 in the left panel for GII.3-GII.6 panel.
2	P19-d874_c	Lane 2 in the left panel for GII.3-GII.6 panel.
3	GII.3 Gro_2014	Lane 3 in the left panel for GII.3-GII.6 panel.
4	GII.3 MX_2005	Lane 4 in the left panel for GII.3-GII.6 panel.
5	RLuc	Lane 5 in the left panel for GII.3-GII.6 panel.
6	Ladder	Lane 6 in the left panel for GII.3-GII.6 panel.
7	GII.6_2010	Lane 7 in the left panel for GII.3-GII.6 panel.
8	P5-d441_6	Lane 8 in the left panel for GII.3-GII.6 panel.
9	P5-d308_2	Lane 9 in the left panel for GII.3-GII.6 panel.
10	P5-d0_1	Lane 10 in the left panel for GII.3-GII.6 panel.

Full unedited blot for Supplemental Figure 6

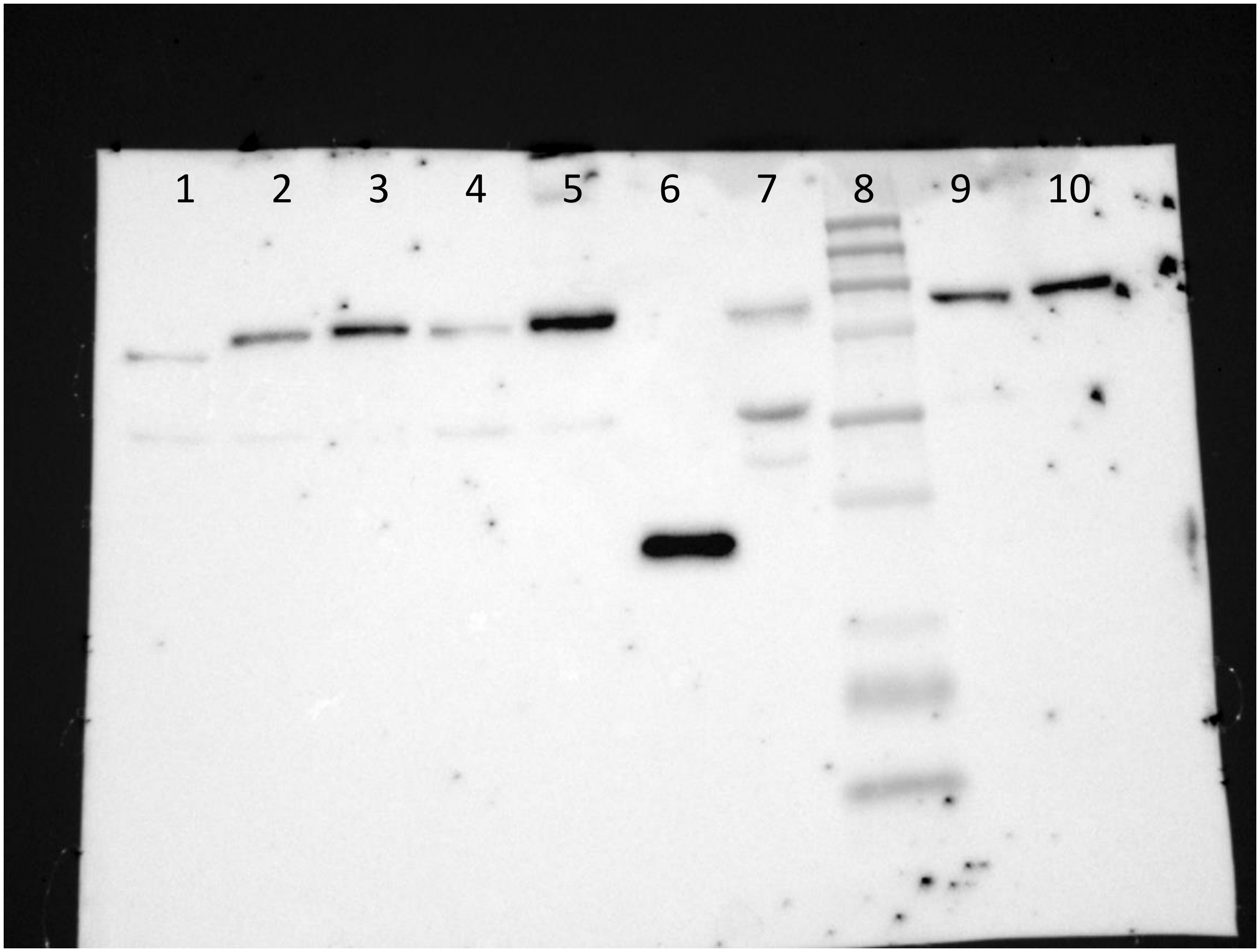

Lane	Protein	Location in Supplemental Figure 6
1	N.A.	Other Rluc-VP1 protein not included in this study.
2	P8-d340_4	Lane 1 of the middle panel of GII.4 blots.
3	P13-d0_3	Lane 2 of the middle panel of GII.4 blots.
4	P13-d51_8	Lane 3 of the middle panel of GII.4 blots.
5	P15-d174_1	Lane 4 of the middle panel of GII.4 blots.
6	RLuc	Lane 5 of the middle panel of GII.4 blots.
7	GII.4 Sy 2012	Lane 6 of the middle panel of GII.4 blots.
8	Ladder	Lane 7 of the middle panel of GII.4 blots.
9	P17-d0_1	Lane 8 of the middle panel of GII.4 blots.
10	P17-d384_1	Lane 9 of the middle panel of GII.4 blots.

Full unedited blot for Supplemental Figure 6

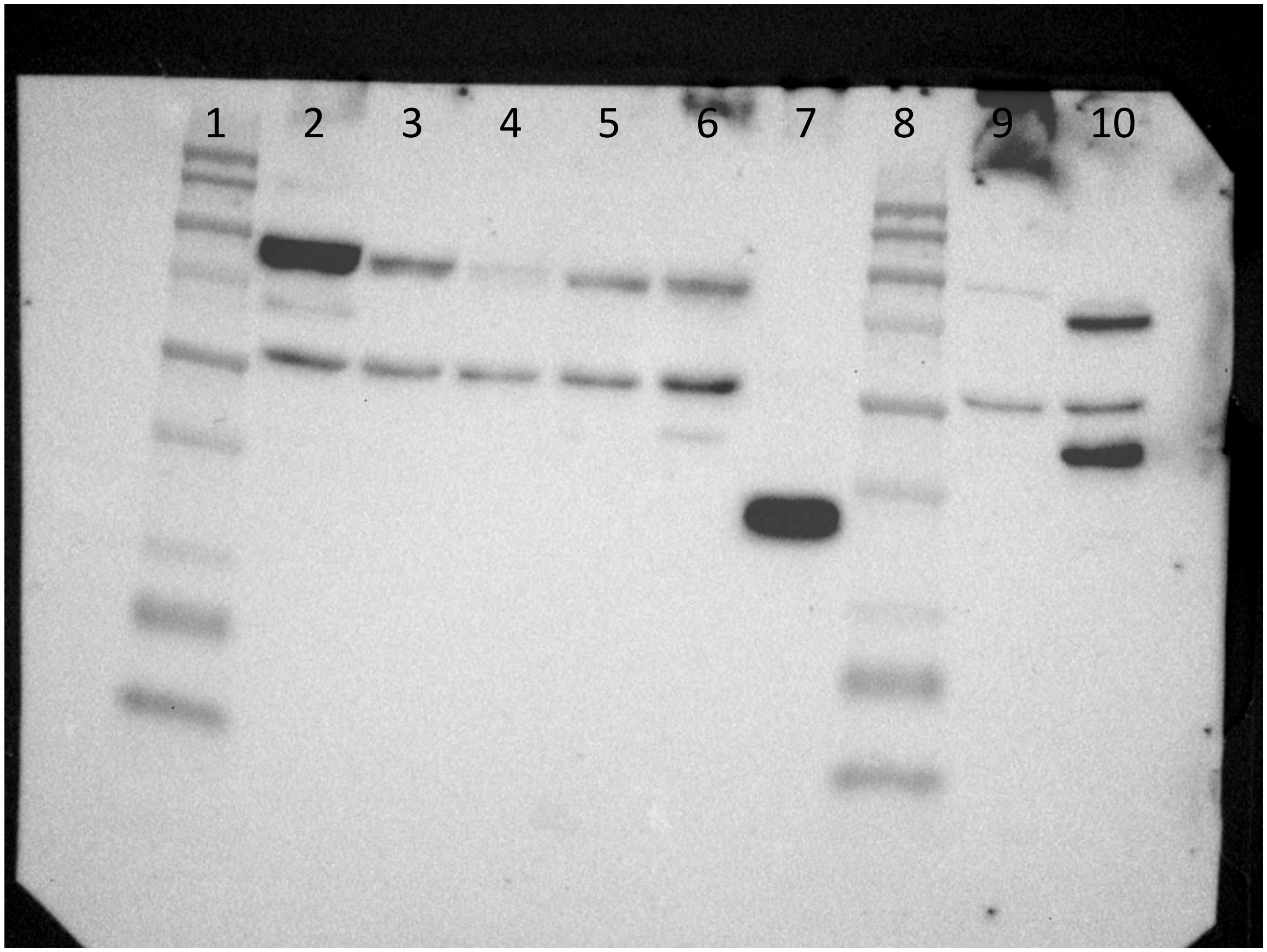

Lane	Protein	Location in Supplemental Figure 6
1	Ladder	Lane 1 of the right panel of GII.4 blots.
2	P9-d0_1	Lane 2 of the right panel of GII.4 blots.
3	P9-d176_5	Lane 3 of the right panel of GII.4 blots.
4	P3-d72_4	Lane 4 of the right panel of GII.4 blots.
5	GII.4 NO 2009	Lane 5 of the right panel of GII.4 blots.
6	GII.4 Sy 2012	Lane 6 of the right panel of GII.4 blots.
7	Rluc	Lane 7 of the right panel of GII.4 blots.
8	Ladder	Not included in panel.
9	N.A.	Other Rluc-VP1 protein not included in this study.
10	N.A.	Other Rluc-VP1 protein not included in this study.

Full unedited blot for Supplemental Figure 6

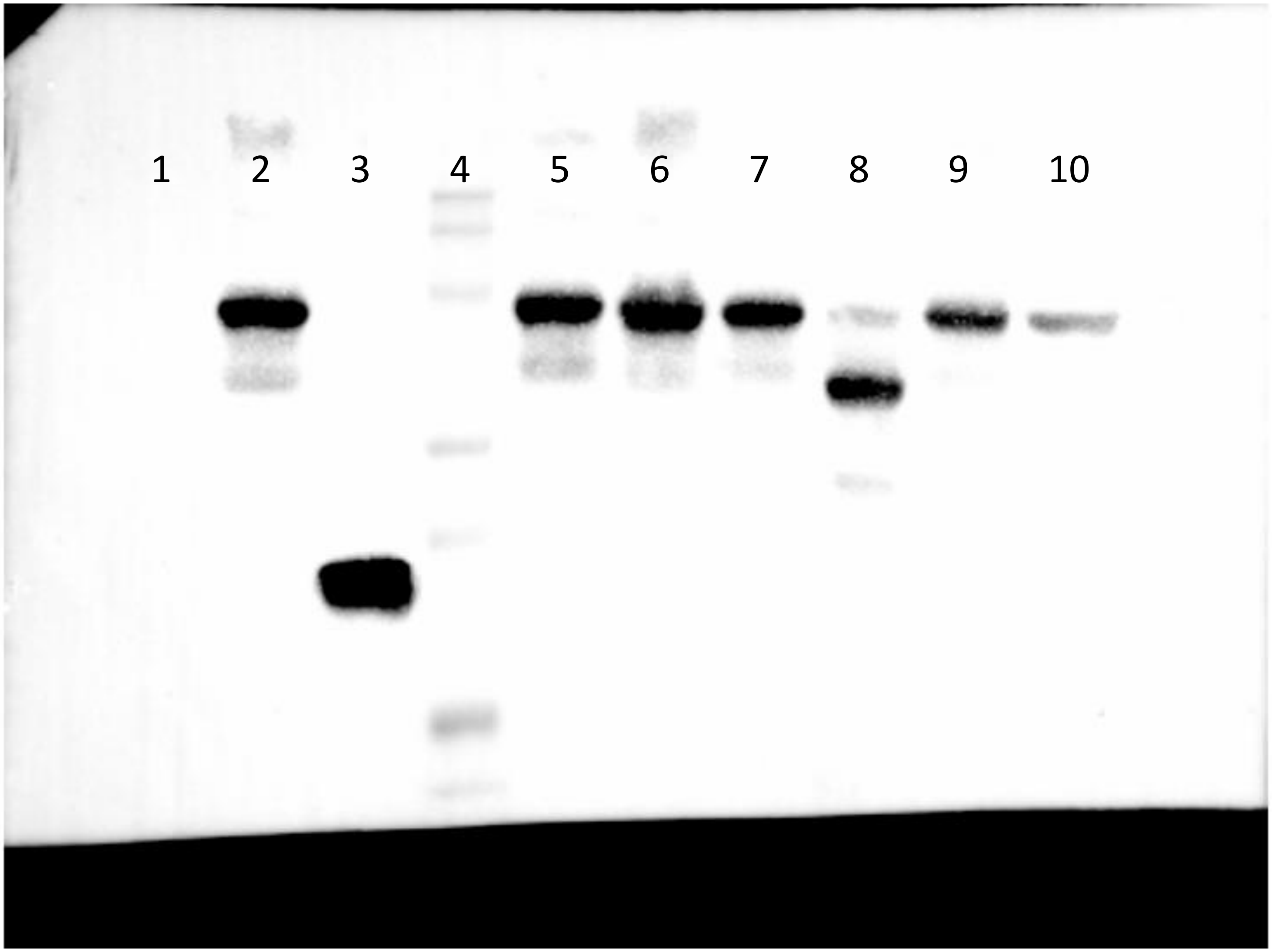

Lane	Protein	Location in Supplemental Figure 6
1	GFP	Not included in the panel.
2	GII.4 Sy 2012	Lane 9 of the GII.14 blot.
3	RLuc	Lane 8 of the GII.14 blot.
4	Ladder	Lane 7 of the GII.14 blot.
5	GII.14_2007	Lane 6 of the GII.14 blot.
6	P18-d1815_c	Lane 5 of the GII.14 blot.
7	P18-d1273_c	Lane 4 of the GII.14 blot.
8	P18-d1138_c	Lane 3 of the GII.14 blot.
9	P18-d757_2	Lane 2 of the GII.14 blot.
10	P18-d167_1	Lane 1 of the GII.14 blot.

Full unedited blot for Supplemental Figure 6

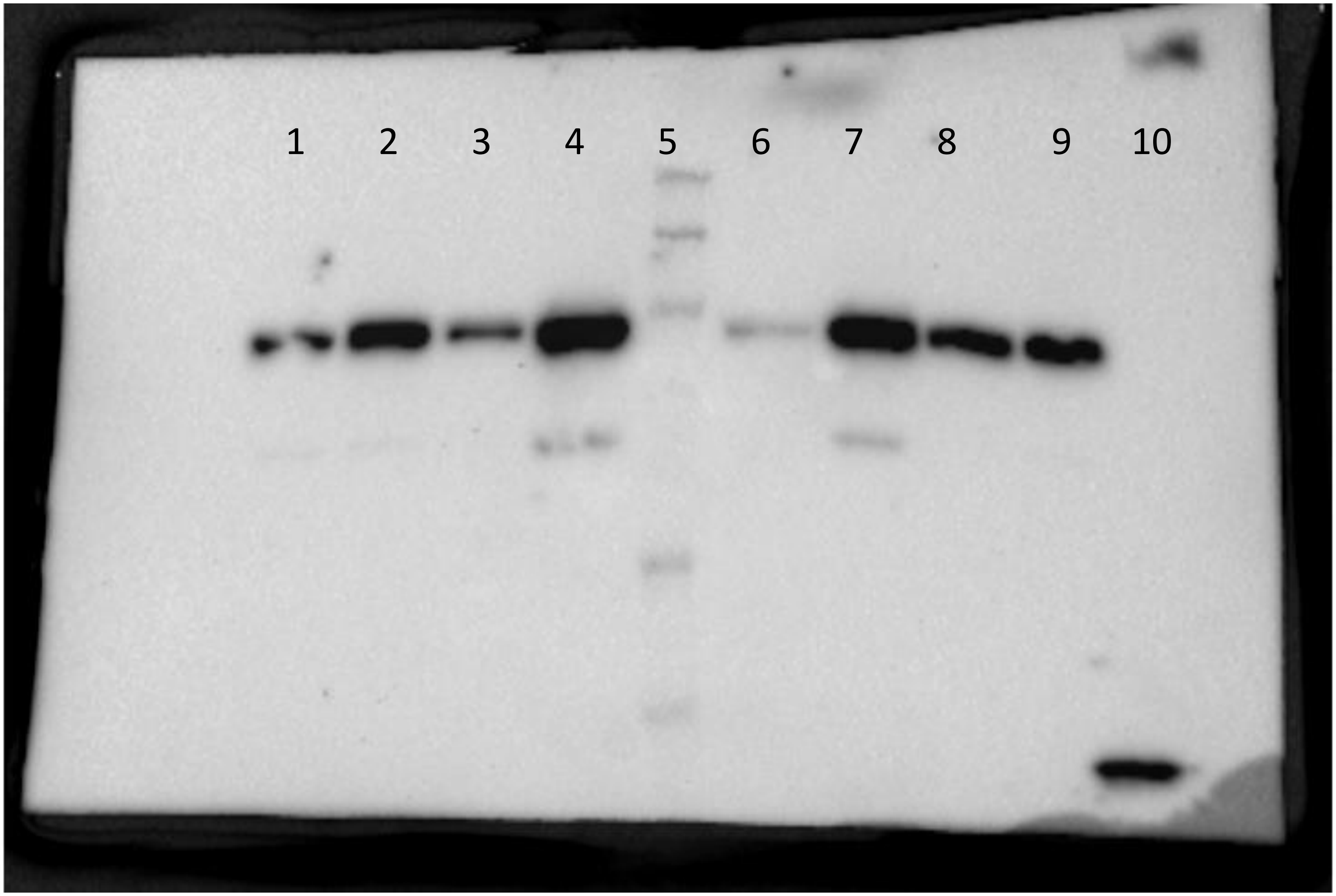

Lane	Protein	Location in Supplemental Figure 6
1	P18-d167_1 wt	Lane 1 of the **P18-d167_1 mutants** blot.
2	P18-d167_1 S310T	Lane 2 of the **P18-d167_1 mutants** blot.
3	P18-d167_1 N343I	Lane 3 of the **P18-d167_1 mutants** blot.
4	P18-d167_1 K369Q	Lane 4 of the **P18-d167_1 mutants** blot.
5	Ladder	Lane 5 of the **P18-d167_1 mutants** blot.
6	P18-d167_1 S310T N343I	Lane 6 of the **P18-d167_1 mutants** blot.
7	P18-d167_1 S310T K369Q	Lane 7 of the **P18-d167_1 mutants** blot.
8	P18-d167_1 N343I K369Q	Lane 8 of the **P18-d167_1 mutants** blot.
9	P18-d167_1 S310T N343I K369Q	Lane 9 of the **P18-d167_1 mutants** blot.
10	RLuc	Lane 10 of the **P18-d167_1 mutants** blot.

Full unedited blot for Supplemental Figure 6

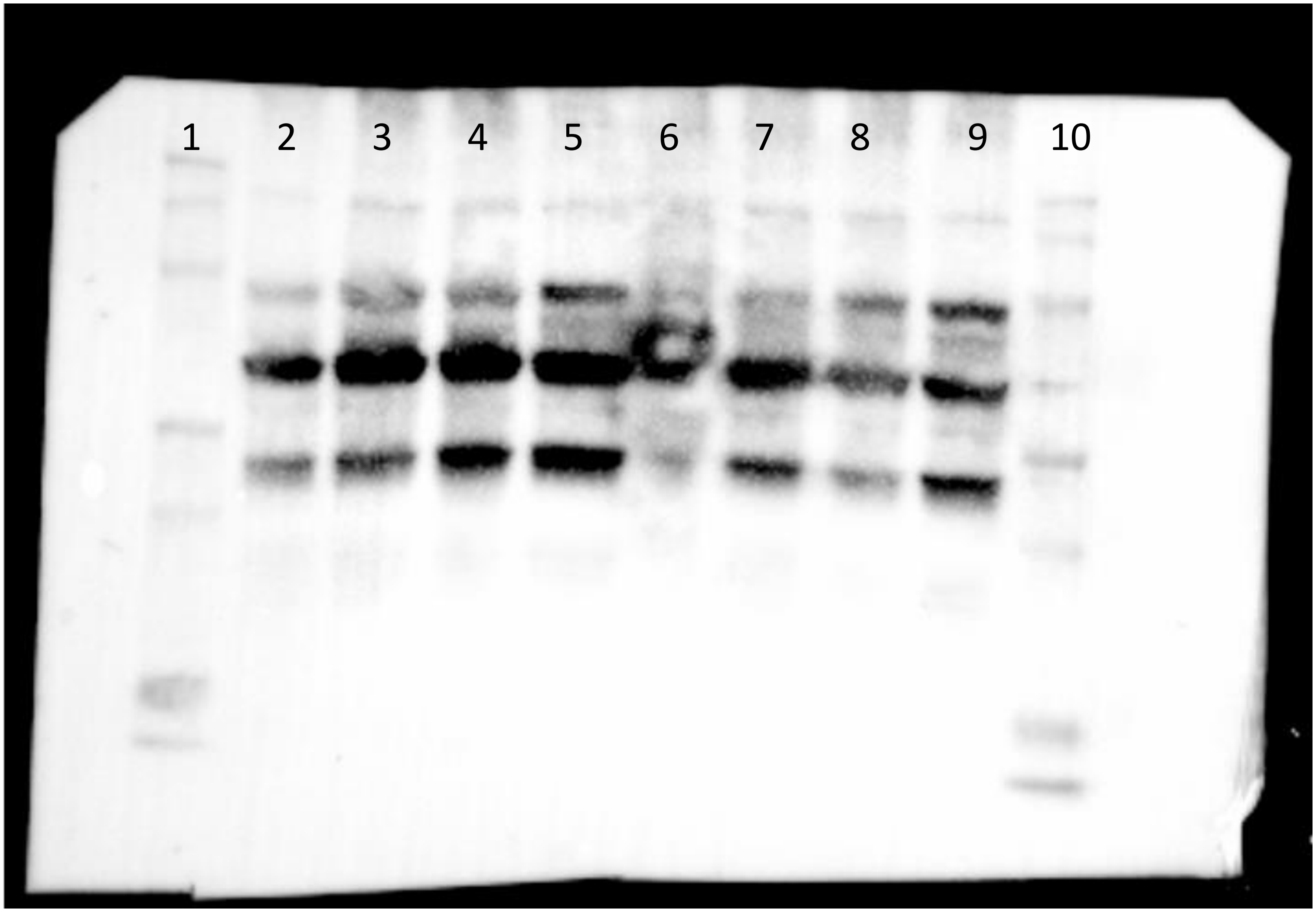

Lane	Protein	Location in Supplemental Figure 6
1	Ladder	Lane 1 of the P18-d1138_c mutants blot.
2	P18-d1138_c wt	Lane 2 of the P18-d1138_c mutants blot.
3	P18-d1138_c T310S	Lane 3 of the P18-d1138_c mutants blot.
4	P18-d1138_c I343N	Lane 4 of the P18-d1138_c mutants blot.
5	P18-d1138_c Q369K	Lane 5 of the P18-d1138_c mutants blot.
6	P18-d1138_c T310S I343N	Lane 6 of the P18-d1138_c mutants blot.
7	P18-d1138_c T310S Q369K	Lane 7 of the P18-d1138_c mutants blot.
8	P18-d1138_c I343N Q369K	Lane 8 of the P18-d1138_c mutants blot.
9	P18-d1138_c T310S I343N Q369K	Lane 9 of the P18-d1138_c mutants blot.
10	Ladder	Lane 10 of the P18-d1138_c mutants blot.

Full unedited blot for Supplemental Figure 6

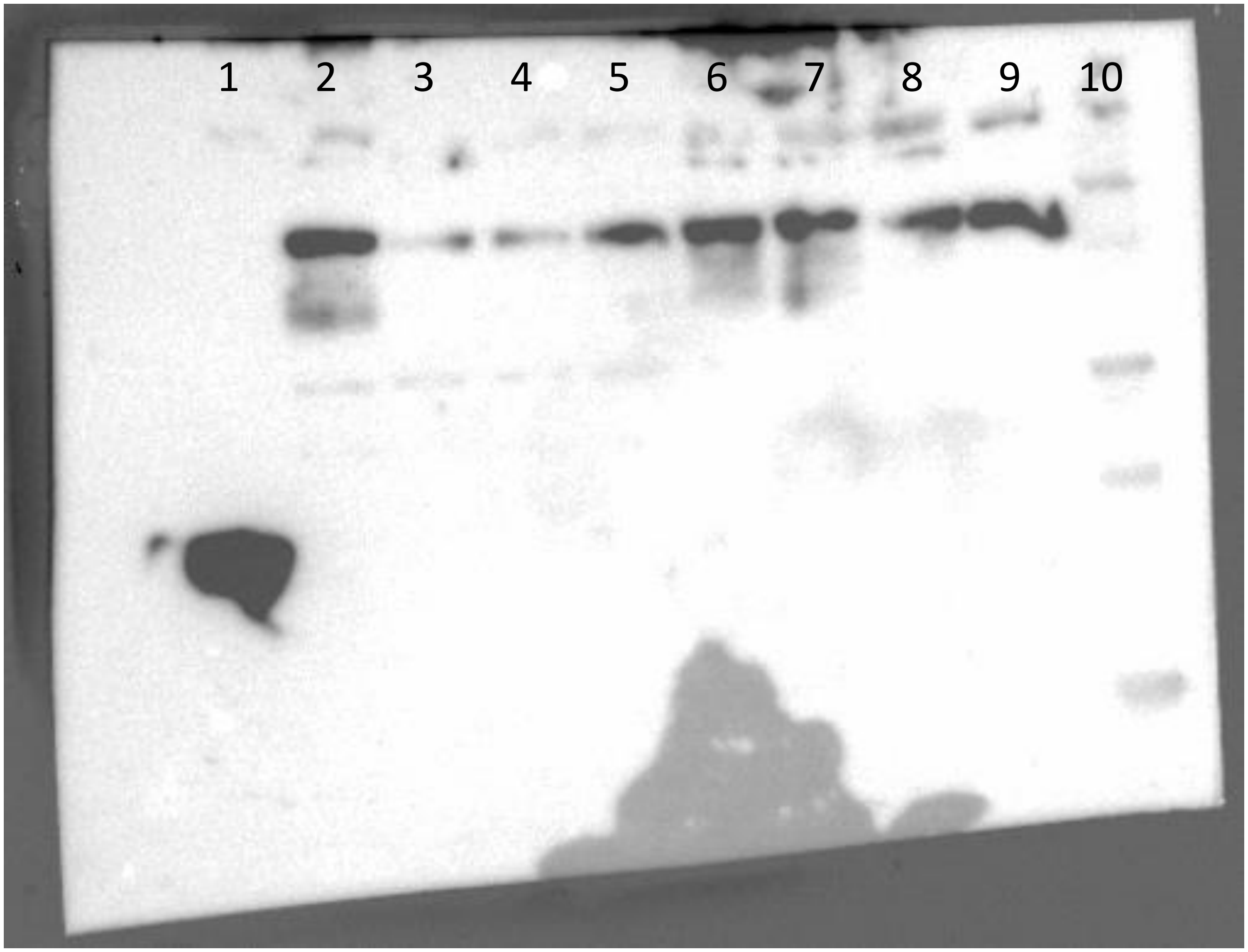

Lane	Protein	Location in Supplemental Figure 6
1	RLuc	Lane 10 of the GII.14_2007 mutants blot.
2	GII.14_2007 S310T N343I K369Q	Lane 9 of the GII.14_2007 mutants blot.
3	GII.14_2007 N343I K369Q	Lane 8 of the GII.14_2007 mutants blot.
4	GII.14_2007 S310T K369Q	Lane 7 of the GII.14_2007 mutants blot.
5	GII.14_2007 S310T N343I	Lane 6 of the GII.14_2007 mutants blot.
6	GII.14_2007 K369Q	Lane 5 of the GII.14_2007 mutants blot.
7	GII.14_2007 N343I	Lane 4 of the GII.14_2007 mutants blot.
8	GII.14_2007 S310T	Lane 3 of the GII.14_2007 mutants blot.
9	GII.14_2007 wt	Lane 2 of the GII.14_2007 mutants blot.
10	Ladder	Lane 1 of the GII.14_2007 mutants blot.
